# Prolyl hydroxylase domain protein 3 and asparaginyl hydroxylase factor inhibiting HIF-1 levels are predictive of tumoral behavior and prognosis in hepatocellular carcinoma

**DOI:** 10.18632/oncotarget.14677

**Published:** 2017-01-16

**Authors:** Mingyang Ma, Shuyao Hua, Gang Li, Sumei Wang, Xue Cheng, Songqing He, Ping Wu, Xiaoping Chen

**Affiliations:** ^1^ Hepatic Surgery Center, Tongji Hospital, Tongji Medical College, Huazhong University of Science and Technology, Hubei Province for the Clinical Medicine Research Center of Hepatic Surgery, Key Laboratory of Organ Transplantation, Ministry of Education and Ministry of Public Health, Wuhan 430030, China; ^2^ Department of Pathophysiology, School of Basic Medicine, Tongji Medical College, Huazhong University of Science and Technology, Wuhan 430030, China; ^3^ Department of Surgery, Liyuan Hospital, Huazhong University of Science and Technology, Wuhan 430077, China; ^4^ Department of Hepatobiliary Surgery, The First Affiliated Hospital of Guangxi Medical University, Nanning 530021, China; ^5^ Laboratory of Hepatobiliary and Pancreatic Surgery, Affiliated Hospital of Guilin Medical University, Guilin 541001, China; ^6^ Guangxi Key Laboratory of Molecular Medicine in Liver Injury and Repair, Guilin 541001, China

**Keywords:** hepatocellular carcinoma, hypoxia-inducible factors, prolyl hydroxylase domain-containing proteins, asparaginyl hydroxylase factor inhibiting HIF-1, prognostic factor

## Abstract

Hypoxia-inducible factors (HIFs) are key regulators in oxygen homeostasis. Their stabilization and activity are regulated by prolyl hydroxylase domain (PHD)-1, -2, -3 and factor inhibiting HIF (FIH). This study investigated the relation between these oxygen sensors and the clinical behaviors and prognosis of hepatocellular carcinoma (HCC). Tissue microarray and RT-PCR analysis of tumor tissues and adjacent non-tumor liver tissues revealed that mRNA and protein levels of both PHD3 and FIH were lower within tumors. The lower expression of PHD3 in tumor was associated with larger tumor size, incomplete tumor encapsulation, vascular invasion and higher Ki-67 LI (*p* < 0.05). The lower expression of FIH in tumor was associated with incomplete tumor encapsulation, vascular invasion, as well as higher TNM stage, BCLC stage, microvascular density and Ki-67 LI (*p* < 0.05). Patients with reduced expression of PHD3 or FIH had markedly shorter disease-free survival (DFS), lower overall survival (OS), or higher recurrence (*p* < 0.05), especially early recurrence. Patients with simultaneously reduced expression of PHD3 and FIH exhibited the least chance of forming tumor encapsulation, highest TNM stage (*p* < 0.0083), lowest OS and highest recurrence rate (*p* < 0.05). Multivariate analysis indicated that a lower expression of FIH independently predicted a poor prognosis in HCC. These findings indicate that downregulation of PHD3 and FIH in HCC is associated with more aggressive tumor behavior and a poor prognosis. PHD3 and FIH may be potential therapeutic targets for HCC treatment.

## INTRODUCTION

Hepatocellular carcinoma (HCC) is one of the most common cancers and the third most frequent cause of cancer-related death worldwide [1, 2]. This situation is particularly concerning in China. Statistics have shown that the annual incidence of HCC in China alone contributes to 55% of global HCC cases [3]. Previously, we have conducted a series of clinical investigations involving the traditional hepatic resection and targeted therapy on unresectable HCC patients [4–9]. Unfortunately, most HCC patients present with symptoms at a late advanced stage, at which point the tumor is unresectable and carries a very poor prognosis, and conventional therapy with cytotoxic agents provides a marginal benefit [1]. More importantly, because of its heterogeneity and aggressive nature, full prognostic evaluation and effective systemic therapies for HCC patients are urgently needed [10].

Because the diffusion limit of oxygen in tissue is approximately 100 μm, the rapidly expanding mass of tumor cells becomes inadequately oxygenated [11]. This phenomenon makes hypoxia one of the fundamental micro-environmental features of solid tumors and even in some non-solid tumors, such as leukaemias [12, 13]. Hypoxia plays a critical role in various tumor-related cellular and physiologic events [14, 15]. It has been accepted for a long time that the ability of tumor cells to adapt to a reduced oxygen and nutrient supply is vital for their survival [16]. Hypoxia-inducible factors (HIFs) are the key molecules to maintain oxygen homeostasis and mediate the adaptive responses to reduced oxygen levels in cells, making the cells capable of surviving under a hypoxic microenvironment. Many recent studies have provided convincing evidence of a strong correlation between the activated HIF-1 pathway and tumor metastasis, angiogenesis, and poor patient prognosis as well as tumor resistance therapy [17–19]. In one of our *in vivo* study with H22 cell-bearing mice model, increased HIF expression and one of its downstream event, angiogenesis, were both observed [20].

Given that HIFs have very wide range of transcriptional targets, more than 100 direct target genes of HIF-1 have been uncovered till now [21], the intricate regulation process of HIF has gained increasing attention. As a heterodimer, HIFs are composed of an oxygen-regulated α subunit and a constitutively expressed β subunit. It is well known that its stability is regulated, at the post-translation level, by oxygen-sensing HIF prolyl hydroxylases, also named prolyl hydroxylase domain-containing (PHD) proteins. Site-special hydroxylation by PHDs enables HIF-α binding with VHL tumor suppression protein and subsequently undergoing proteasomal degradation by ubiquitation. Under hypoxia, the enzymatic activity of PHDs is inhibited, leading to the accumulation of HIF-α, which then is dimerized with HIF β and translocates into the nucleus to activate its transcription of target genes. In human, three different subtypes of PHDs have been identified, which had conserved COOH-terminal regions responsible for hydroxylase activity but different N terminus and hydroxylation sites [22, 23]. In the meanwhile, the transcriptional activity of HIF could be controlled by asparaginyl hydroxylase factor inhibiting HIF-1 (FIH). By hydroxylase on Asp803 of the HIF-1 C-terminal transactivation domain, the ability of HIF-1 binding to the transcriptional coactivator p300/CBP in the nucleus was inhibited. With these two hydroxylation processes, the HIF pathway could be effectively repressed by either the destruction or inactivation of HIF-α in well-oxygenated cells but activated in hypoxia cells [24].

HIF hydroxylases were recently recognized as important players in cancer biology by interfering with angiogenesis and metastasis, such as in prostate cancer [25], breast cancer [26], colorectal cancer (CRC) [27], and renal cell carcinoma [28]. Interestingly, in recent years, several studies on different tumor type, *in vivo* or *in vitro*, demonstrated inconsistent data of the expression pattern, subcellular distribution, as well as the prognostic value of these enzymes [29–31], suggesting that they may have diverse effects on the basis of tumor type.

Unfortunately, few clinical studies on the expression level of PHDs or FIH had been conducted in HCC patients. Additionally, several questions need to be answered in HCC, such as how these hydroxylases are expressed, what is the correlation among different types of hydroxylases, HIF-1 α and clinical behaviors, and whether they can influence the prognosis of HCC patients. In the current study, we determined both the mRNA and protein expression of PHD1–3 and FIH in HCC tissue samples by RT-PCR, tissue microarray (TMA) immunohistochemistry (IHC) analysis and western blotting assay, and correlated the different expression levels of these hydroxylases to the clinical and histoprognostic characteristics and prognosis of HCC patients.

## RESULTS

### Patient characteristics

The demographic, clinical and histopathological data for 81 patients with HCC are presented in Supplementary Table 1. The majority of the study population was male (90.12%) with a median age of 47 years (range: 27–75 years). The median follow-up time of the sur*vivo*rs was 41 months (range: 3–62 months). Eighty-eight percent of the patients had been diagnosed with liver cirrhosis. Approximately 89% of the patients had HBV, but only 1 case had HCV. Based on the TNM classification of malignant tumors, 43 patients were categorized as stage I to II, and 38 were categorized as stage III to IV. Recurrence occurred in 49 patients (60.49%) during the follow-up. The tumor recurrence in most of the cases occurred in the liver (47 cases); there was one metastasis in the lung and one in both the liver and lung.

### Expression of PHD1 in HCC patients

To explore and compare the different expression levels of four HIF hydroxylases in HCC tumor tissue and ANLTs, IHC on TAMs and western blotting were conducted. IHC analysis showed that PHD1 was predominantly cytoplasmic, although nuclear staining was observed. Neither the median score of PHD1 nor the percentage of PHD1(+) (indicating higher expression) in tumor tissues was obviously different from that in paired ANLTs in all 81 cases (*p* = 0.5196, Wilcoxon signed-rank test; *p* = 0.7532, χ^2^ test), as shown in Supplementary Table 2 and Supplementary Figure 1A and 1B. Consistently, western blotting of 24 randomly collected cases also indicated that there was no significant difference in the relative PHD1 protein level between the tumor tissue and ANLTs (*p* = 0.5427, paired *t-test*) (Supplementary Figure 1C and 1D).

Moreover, we performed a comparison of the PHD1 mRNA in 40 randomly selected pairs of tumor tissues and their ANLTs. The relative expression of PHD1 was normalized to the expression of the endogenous gene GAPDH. According to our classification criteria, ΔΔCt ≤ –1 or ≥ 1, PHD1 in tumor tissue was increased in 8 cases and decreased in 16 cases. However, in 16 cases, no obvious change could be detected. The box plot analysis visualized the ΔCt distribution and indicated that the PHD1 mRNA level showed no difference between the tumor tissue and ANLTs (*p* = 0.0635, paired *t* test) (Supplementary Figure 1E and 1F).

### Expression of PHD2 in HCC patients

Similar to the result concerning PHD1, IHC analysis also showed that PHD2 was predominantly cytoplasmic, although nuclear staining was observed. There was no significant difference between the median score of PHD2 or the percentage of PHD2(+) in tumor tissues and paired ANLTs (*p* = 0.4477, Wilcoxon signed rank test; *p* = 0.5152, χ^2^ test), as shown in Supplementary Table 2 and Supplementary Figure 2A to 2B. By contrast, when detected by western blotting analysis, tumor tissue presented a considerably higher PHD2 protein level than ANLTs (*p* = 0.0224, paired *t-test*).

Results from real-time PCR indicated that PHD2 in tumor tissue was increased in 8 cases and decreased in 11 cases. However, in 21 cases, no obvious change could be detected (Supplementary Figure 2E). The box plot analysis showed no difference in the ΔCt value between tumor tissue and ANLT (*p* = 0.6772, paired *t* test) (Supplementary Figure 2F).

### Expression of PHD3 in HCC patients

**Figure 1:**
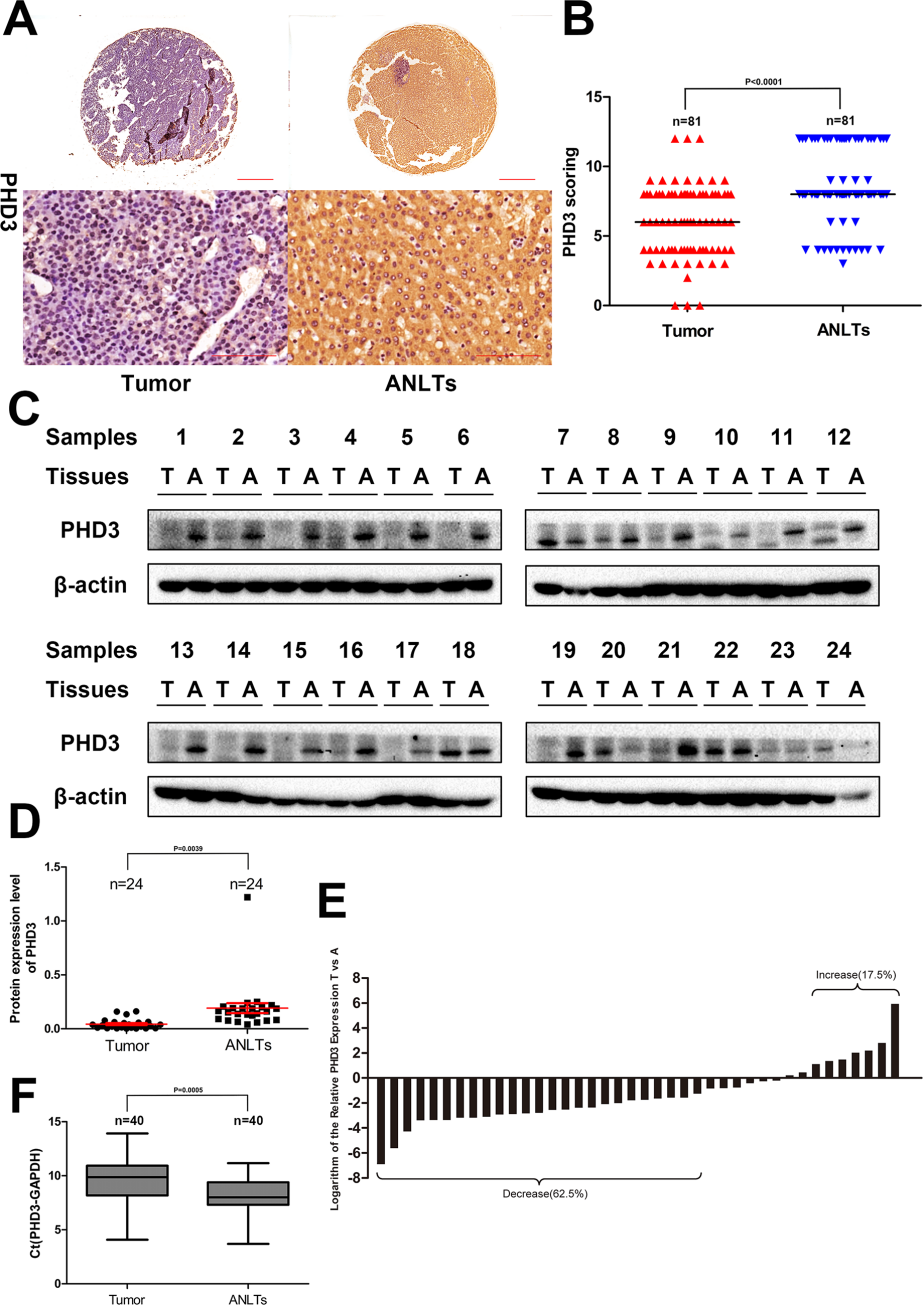
Expression of PHD3 in HCC patients (**A**) IHC analysis of PHD3 expression in 81 HCC tissues and paired ANLTs. Representative images were taken. Scale bar, 500 μm (upper) or 100 μm (lower). (**B**) IHC scoring was displayed by a scatter plot, with the median indicated. (**C**) Western blotting of tumor tissue and paired ANLTs in 24 randomly selected HCC cases with β actin as the loading control. (**D**) The relative densities of PHD3 protein in 24 cases were calculated and shown by a scatter plot, with the mean and SEM indicated. (**E**) the mRNA level of PHD3 was analysed by Real-time PCR in 40 randomly selected HCC tissues and paired ANLTs. The vertical axis means the logarithm base 2 of the relative expression of PHD3 in tumor tissue compared with ANLTs. Bar value ≤ –1 and ≥ 1 indicate that the expression of PHD3 is decreased and increased in tumors, respectively. (**F**) The ΔCt value was presented with a box plot reporting the median values and the interquartile range.

From Figure 1A, it can be observed that PHD3 was predominantly cytoplasmic, although nuclear staining was observed. The median score for PHD3 in tumor tissues was much lower than that in ANLTs, 6 vs. 8 (*p <* 0.0001, Wilcoxon signed rank test). A PHD3(–) tumor was considered in 30 cases, with 12 in ANLTs (*p* = 0.0013, χ^2^ test), as shown in Supplementary Table 2 and Figure 1A to 1B. Similar to what was found in IHC, western blotting detected that the PHD3 level was markedly degraded in tumor tissue (21 in 24 cases, *p* = 0.0039), shown in Figure 1C and 1D.

Regarding PHD3 mRNA expression in tumor, it was increased in 7 cases, decreased in 25 cases and no changing in 8 cases (Figure 1E). We further displayed the distribution of ΔCt in Figure 1F. The result indicated that ΔCt in tumor tissue was significantly higher than that in ANLTs (*p* = 0.0005, paired *t* test) (Supplementary Figure 2C and 2D) indicating that PHD3 mRNA expression was down-regulated in HCC.

### Expression of FIH in HCC patients

**Figure 2:**
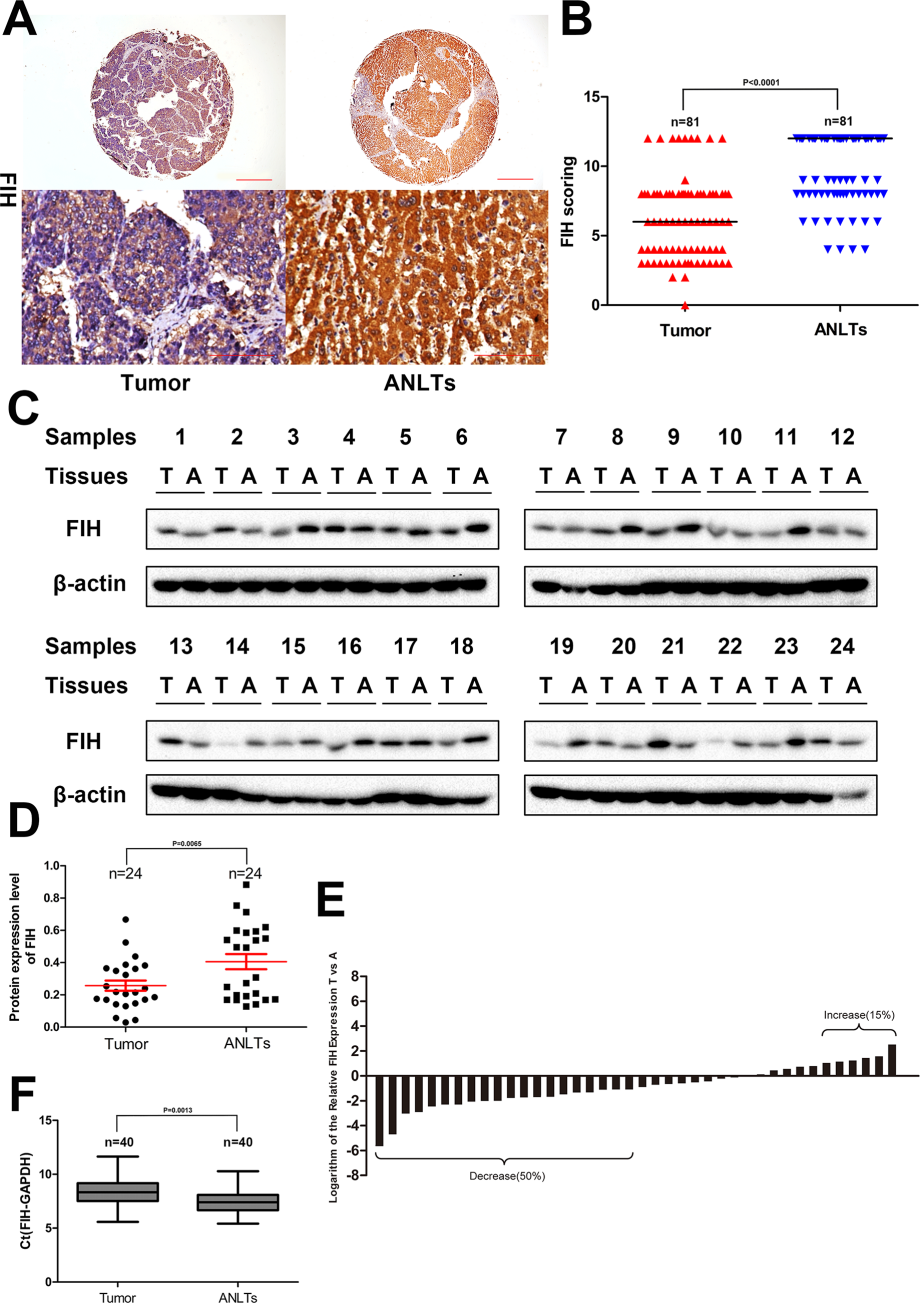
Expression of FIH in HCC patients (**A**) IHC analysis of FIH expression in 81 HCC tissues and paired ANLTs. Representative images were taken. Scale bar, 500 μm (upper) or 100 μm (lower). (**B**) IHC scoring was displayed by a scatter plot, with the median indicated. (**C**) Western blotting of tumor tissue and paired ANLTs in 24 randomly selected HCC cases with β actin as the loading control. (**D**) The relative densities of FIH protein in 24 cases were calculated and shown by a scatter plot, with the mean and SEM indicated. (**E**) the mRNA level of FIH was analysed by Real-time PCR in 40 randomly selected HCC tissues and paired ANLTs. The vertical axis means the logarithm base 2 of the relative expression of FIH in tumor tissue compared with ANLTs. Bar value ≤ –1 and ≥ 1 indicate that the expression of FIH is decreased and increased in tumors, respectively. (**F**) The ΔCt value was presented with a box plot reporting the median values and the interquartile range.

Unlike breast carcinoma or clear cell renal cell carcinoma, in which FIH staining was predominantly nuclear [28, 32], in current study, the staining was mainly in cytoplasm, but very weak in the nucleus. The median score for FIH in TAMs from tumor tissue was 6, which was much lower than the median score of 12 in ANLTs as seen in Supplementary Table 2 and Figure 2A, 2B (*p <* 0.0001, Wilcoxon signed rank test). In all cases, FIH(+) was detected in 59.30% of tumor tissue, significantly lower than 95.10% detected in ANLTs (*p <* 0.0001, Fisher's exact test). The results from IHC were further confirmed by western blotting (Figure 2C). The mean value of the relative density of FIH protein was 0.2573 in tumor but 0.4058 in ANTLs (*p* = 0.0065) (Figure 2D).

At the transcriptional level, FIH in tumor tissue was up-regulated in six cases, down-regulated in 20 cases, and no obvious change in 14 cases (Figure 2E). The box plot displayed a much higher level of distribution of ΔCt value which meant lower expression of FIH in tumor tissue (*p* = 0.0013, compared with ANTLs; paired *t* test) (Figure 2F).

### Correlation of the PHD3 and FIH expression with the clinicopathological characteristics of HCC patients

After it was confirmed that both the mRNA and protein levels of PHD3 and FIH were obviously reduced in tumor tissue, we were notably interested in whether these changes had any correlation with the clinicopathological features of HCC patients. As seen in Table 1, the following variables: gender, age, AFP level, GGT level, tumor size, ALT level, HBV, HCV, cirrhosis, tumor encapsulation, number, invasion, distant metastasis, differentiation, TNM stage, BCLC stage, adjuvant TACE, microvascular density (MVD) and Ki-67 were chosen. Pearson χ^2^ test or Fisher exact tests revealed that PHD3(–) was significantly associated with larger tumor size (*p* = 0.009), none tumor encapsulation (*p* = 0.012), vascular invasion (*p* = 0.041) or higher Ki-67 Li (*p* = 0.006). At the same time, FIH(–) showed correlation with none tumor encapsulation (*p* = 0.018), vascular invasion (*p* = 0.048), higher TNM stage (*p* = 0.003), higher BCLC stage (*p* = 0.015), higher MVD (*p* = 0.009) and higher Ki-67 LI (*p* = 0.020).

**Table 1:** Correlation between the PHD3 and FIH expression levels in TAMs and the clinicopathologic characteristics in HCC patients (n = 81)

Clinicopathological variables	Total	PHD3		*χ*^2^	*p*-value	FIH		*χ*^2^	*p*-value
		Low	High			Low	High		
**Gender**									
Male	73	26	47		0.460*	29	44		0.710*
Female	8	4	4	4		4			
**Age**									
≤ 50	45	17	28	0.024	0.877	18	27	0.023	0.879
> 50	36	13	23			15	21		
**AFP (µg/l)**									
≤ 20	16	5	11	0.286	0.593	4	12		0.256*
> 20	65	25	40			29	36		
**GGT (U/l)**									
≤ 54	31	8	23	2.716	0.099	10	21	1.497	0.221
> 54	50	22	28			23	27		
**ALT (ng/ml)**									
≤ 75	65	26	39		0.388*	28	37	0.744	0.388
> 75	16	4	12			5	11		
**HBV**									
Negative	9	4	5		0.720*	3	6		0.731*
Positive	72	26	46			30	42		
**HCV**									
Negative	80	30	50		1.000*	33	47		1.000*
Positive	1	0	1			0	1		
**Cirrhosis**									
No	10	4	6		1.000*	4	6		1.000*
Yes	71	26	45			29	42		
**Tumor size (cm)**									
≤ 5	31	6	25	6.733	**0.009**	10	21	1.497	0.221
> 5	50	24	26			23	27		
**Tumor encapsulation**									
None	34	18	16	6.356	**0.012**	19	15	5.564	**0.018**
Complete	47	12	35			14	33		
**Tumor number**									
Single	61	22	39	0.100	0.752	22	39	2.237	0.135
Multiple	20	8	12			11	9		
**Vascular invasion**									
No	52	15	37	4.179	**0.041**	17	35	3.897	**0.048**
Yes	29	15	14			16	13		
**Distant metastasis**									
No	80	30	50		1.000*	33	47		1.000*
Yes	1	0	1			0	1		
**Differentiation**									
I–II	56	20	36	0.136	0.712	19	37	3.488	0.062
III–IV	25	10	15			14	11		
**TNM stage**									
I–II	43	12	31	3.276	0.070	11	32	8.725	**0.003**
III–IV	38	18	20			22	16		
**BCLC stage**									
0 + A	45	13	32	2.883	0.090	13	32	5.891	**0.015**
B + C	36	17	19			20	16		
**Adjuvant TACE**									
No	75	28	47		1.000*	30	45		0.683*
Yes	6	2	4			3	3		
**MVD (per HPF)**									
< 45	46	14	32	1.990	0.158	13	33	6.868	**0.009**
≥ 45	35	16	19			20	15		
**Ki-67 LI**									
< 5%	32	6	26	7.586	**0.006**	8	24	5.429	**0.020**
≥ 5%	49	24	25			25	24		

*Fisher's exact test; Statistically significant (*p <* 0.05); Ki-67 LI, Ki-67 labeling index.

The above results indicated that PHD3(–) or FIH(–) correlated with a more aggressive degree of HCC. Next, we divided all of the patients into the following groups according to both the PHD3 and FIH expression status in TAMs, as PHD3(+)/FIH(+), PHD3(–)/FIH(+), PHD3(+)/FIH(–) and PHD3(–)/FIH(–). Comparison between patients in the PHD3(+)/FIH(+) group and PHD3(–)/FIH(–) group was performed by χ^2^ test with the Bonferroni correction, as seen in Supplementary Table 3, indicating that PHD3(–)/FIH(–) patients had an obviously lower chance of forming tumor encapsulation but higher chance of TNM stage than PHD3(+)/FIH(+) patients (*p <* 0.0083). Comparison between multiple groups further confirmed that HCC patients with reduced PHD and FIH simultaneously had more aggressive behaviors.

### Correlation between PHD3 and FIH expression and the prognosis of HCC patients

**Figure 3:**
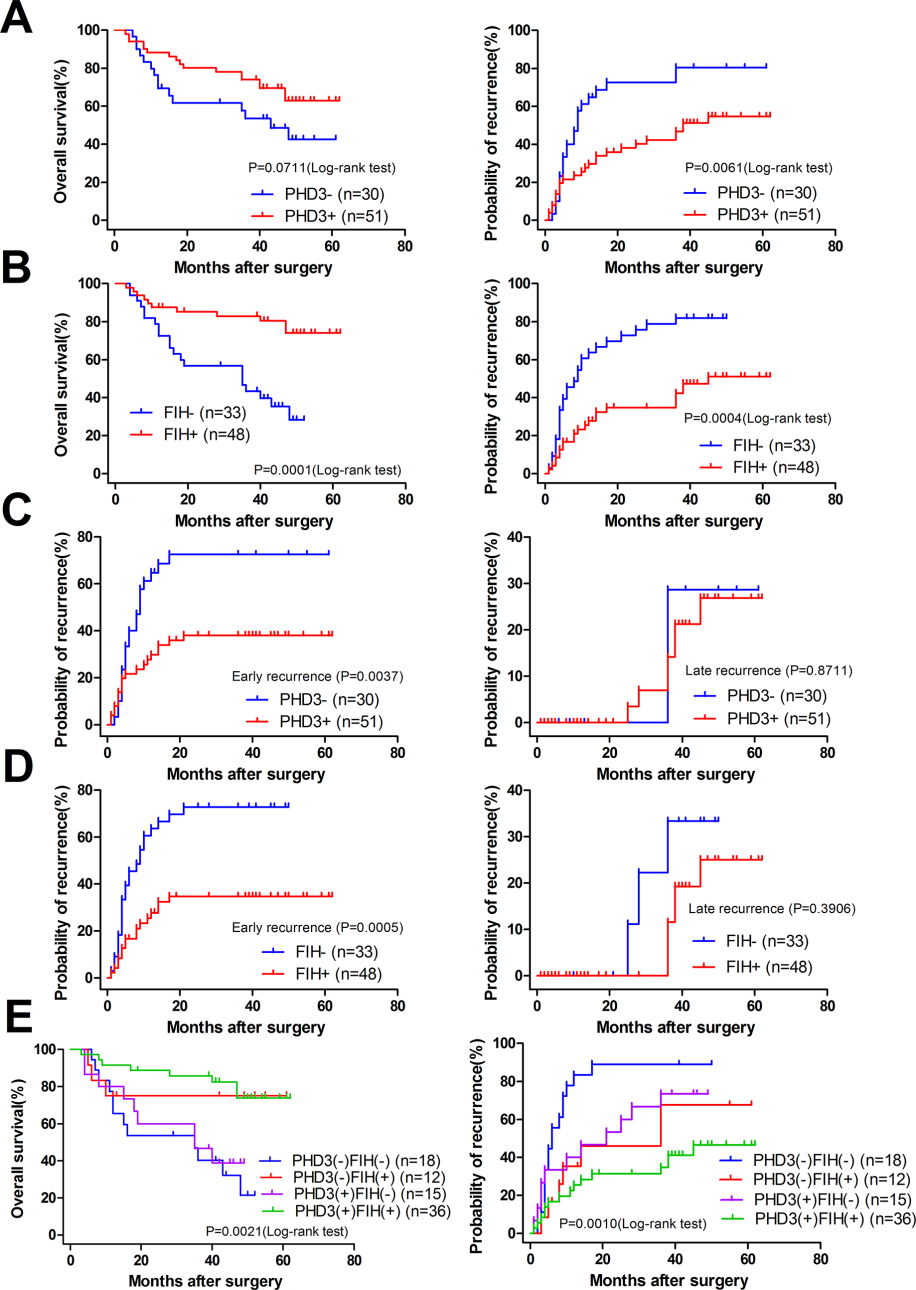
Kaplan-Meier curves for patients with HCC (**A**) OS and RR of patients according to the different expression levels of PHD3 in TAMs. (**B**) OS and RR of patients according to the different expression levels of FIH in TAMs. (**C**) early and late recurrence of patients according to the different expression levels of PHD3 in TAMs. (**D**) early and late recurrence of patients according to the different expression levels of FIH in TAMs. (**E**) OS and RR of patients according to the different expression levels of PHD3 and FIH in TAMs. The log-rank test was used.

We further explored whether the different expression levels of PHD3 or FIH in TAM had a correlation with the prognosis of patients with HCC. As seen in Figure 3A, the log-rank test showed that, compared with PHD3(+) patients, those with PHD3(–) had markedly higher recurrence rate (RR) (80.392% vs. 54.693%, *p* = 0.0061) and a shorter disease-free survival time (DFS, median value of 9 months vs. 38 months; *p <* 0.05), but no significant difference was observed in overall survival (OS) (42.555% vs. 62.901%; *p* = 0.0711). We also found that, in Figure 3B, between patients with FIH(–) and FIH(+), all three indexes were different (28.235% vs. 73.989% for OS, *p* = 0.0001; 9 months vs. 45 months for the median DFS, *p <* 0.05; and 81.818% vs. 51.034% for RR, *p* = 0.0004).

Next, tumor recurrence was classified as early recurrence and late recurrence using 2 years as the cutoff. Figure 3C and 3D indicated that PHD3(–) patients and FIH(–) patients both presented with a higher early-stage recurrence, 72.549% vs. 38.066% (*p* = 0.0037) in PHD3(+) patients and 72.727% vs. 34.712% (*p* = 0.0005) in FIH(+) patients. No difference was found in late-stage recurrence.

When the patients were divided into four groups, according to the expression of both PHD3 and FIH status in TAMs, an obvious difference was found among multiple groups (*p* = 0.0021 in OS and *p* = 0.0010 in RR; log-rank test). Patients with PHD3(–)/ FIH(–) had a higher RR of 88.889% than 46.529% in patients with PHD3(+)/FIH(+). The OS of the former group was as low as 21.429%, while in the latter group was 73.893% (Figure 3E). The Results indicated that PHD3(–)/FIH(–) was associated with a poorest prognosis in patients with HCC.

### Stratified analysis of the prognostic significance of PHD3 or FIH expression in patients with different clinicopathological characteristics

**Figure 4:**
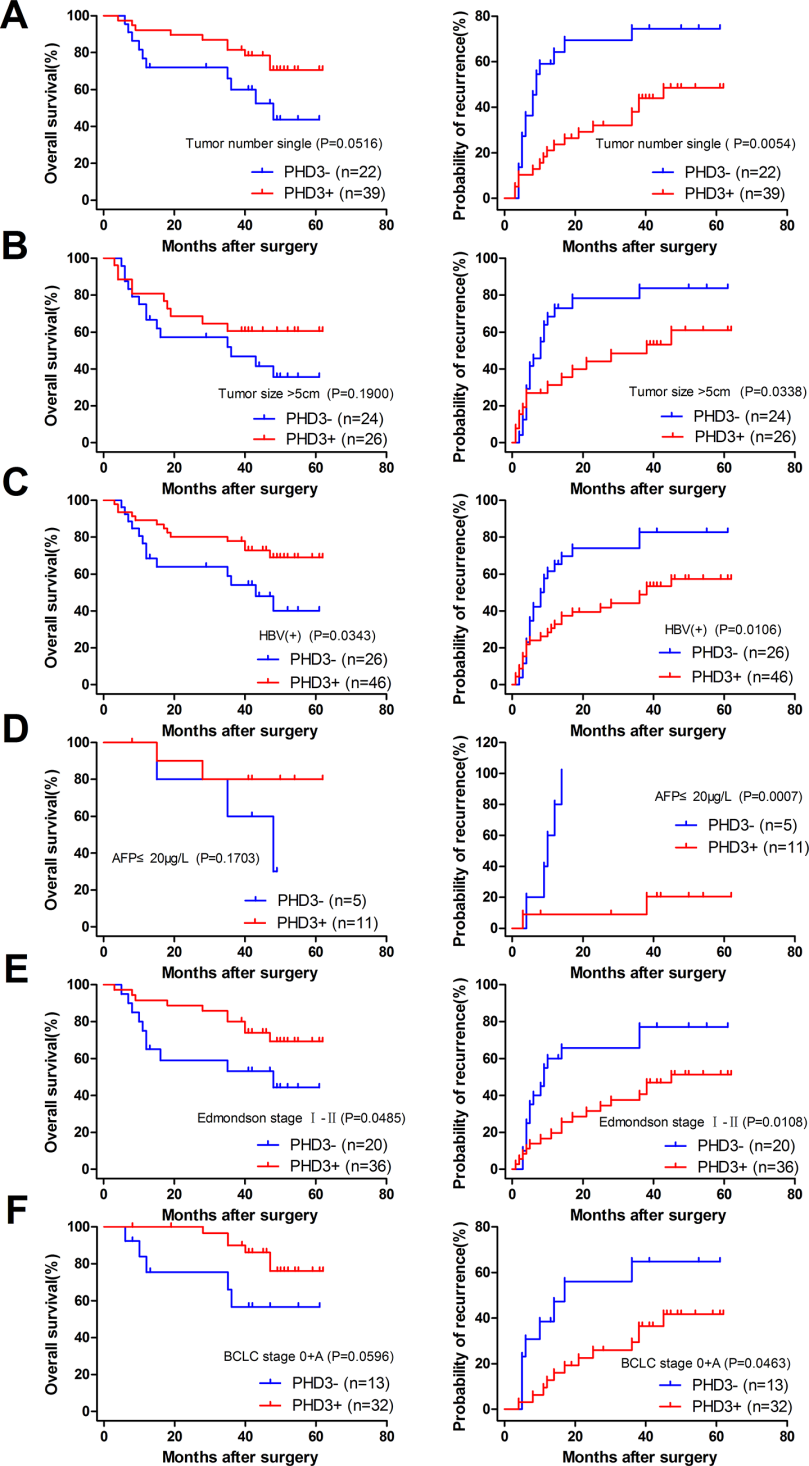
Kaplan-Meier curves for patients with different clinicopathological characteristics according to PHD3 expression level in TAMs (**A**) Prognosis of the patients with a single tumor. OS 43.732% in PHD3(–) vs. 70.595% in PHD3(+), *p* = 0.0516. RR 74.432% in PHD3(–) vs. 48.583% in PHD3(+), *p* = 0.0054. (**B**) Prognosis of the patients with tumor size > 5 cm. OS 35.622% in PHD3(–) vs. 60.577% in PHD3(+), *p* = 0.1900. RR 83.750% in PHD3(–) vs. 60.921% in PHD3(+), *p* = 0.0338. (**C**) Prognosis of the patients with HBV (+). OS 40.071% in PHD3(–) vs. 69.020% in PHD3(+), *p* = 0.0343. RR 82.692% in PHD3(–) vs. 57.358% in PHD3(+), *p* = 0.0106. (**D**) Prognosis of the patients with AFP ≤ 20 μg/l. OS 30.000% in PHD3(–) vs. 80.000% in PHD3(+), *p* = 0.1703. RR 100.000% in PHD3(–) vs. 20.455% in PHD3(+), *p* = 0.0007. (**E**) Prognosis of the patients with Edmondson stage I–II. OS 44.318% in PHD3(–) vs. 69.347% in PHD3(+), *p* = 0.0485. RR 77.143% in PHD3(–) vs. 51.302% in PHD3(+), *p* = 0.0108. (**F**) Prognosis of the patients with BCLC 0+A. OS 56.643% in PHD3(–) vs. 76.103% in PHD3(+), *p* = 0.0596. RR 64.835% in PHD3(–) vs. 41.791% in PHD3(+), *p* = 0.0463. Log-rank test was used.

**Figure 5:**
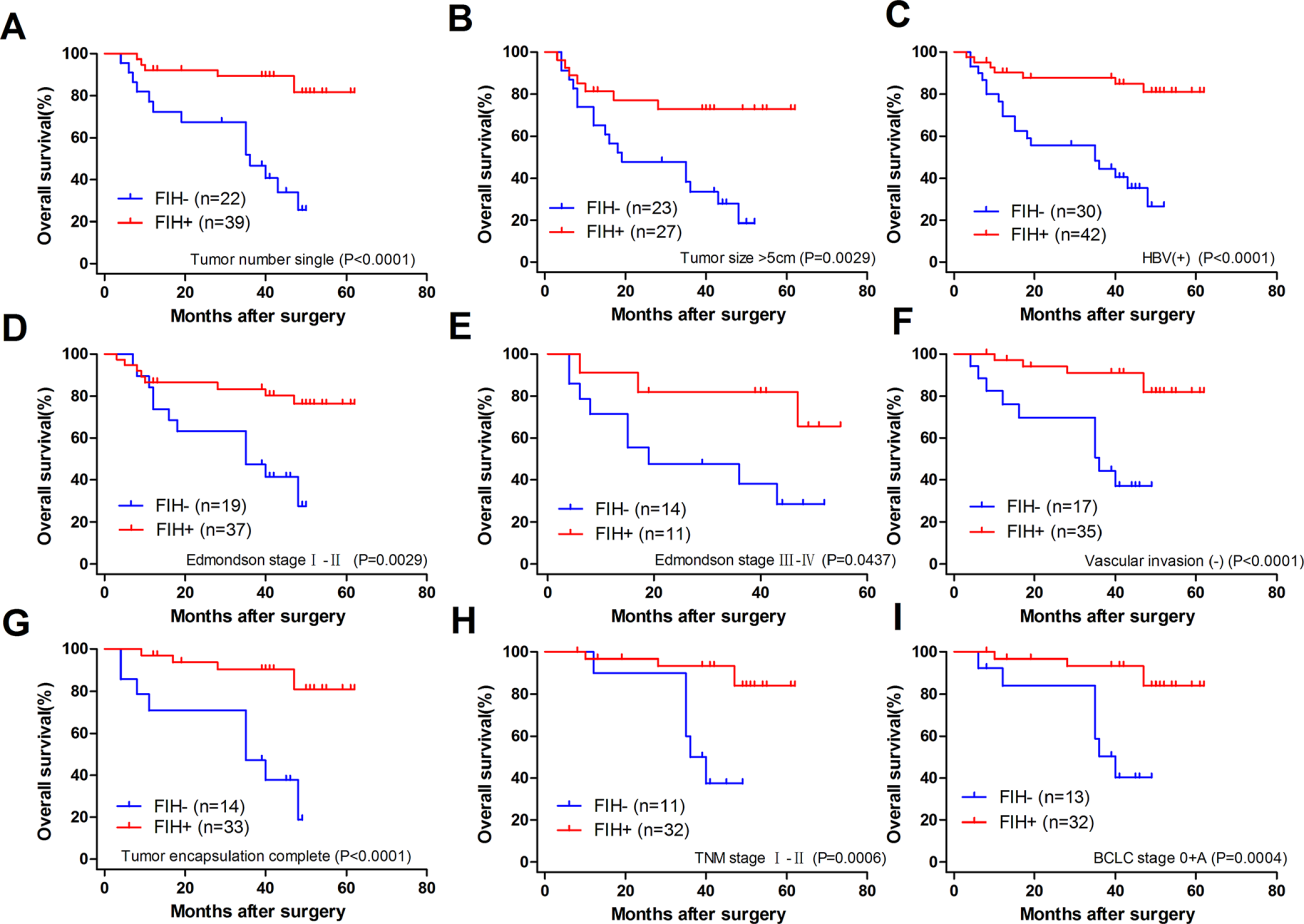
Overall survival Kaplan-Meier curves for patients with different clinicopathological characteristics according to FIH expression level in TAMs (**A**) OS of the patients with single tumor. 25.510% in FIH(–) vs. 81.525% in FIH(+), *p* < 0.0001. (**B**) OS of the patients with tumor size > 5 cm. 18.599% in FIH(–) vs. 72.754% in FIH(+), *p* = 0.0029. (**C**) OS of the patients with HBV (+). 26.561% in FIH(–) vs. 81.080% in FIH(+), *p* < 0.0001. (**D**) OS of the patients with Edmondson stage I–II. OS 27.632% in FIH(–) vs. 76.246% in FIH(+), *p* = 0.0029. (**E**) OS of the patients with Edmondson stage III–IV. 28.571% in FIH(–) vs. 65.455% in FIH(+), *p* = 0.0437. (**F**) OS of the patients with no vascular invasion. 36.953% in FIH(–) vs. 81.802% in FIH(+), *p* < 0.0001. (**G**) OS of the patients with complete tumor encapsulation. 18.857% in FIH(–) vs. 80.875% in FIH(+), *p* < 0.0001. (**H**) OS of TNM I-II stage. 37.500% in FIH(–) vs. 83.986% in FIH(+), *p* = 0.0006. (**I**) OS of the patients with BCLC 0+A. 40.280% in FIH(–) vs. 83.986% in FIH(+), *p* = 0.0004. Log-rank test was used.

**Figure 6:**
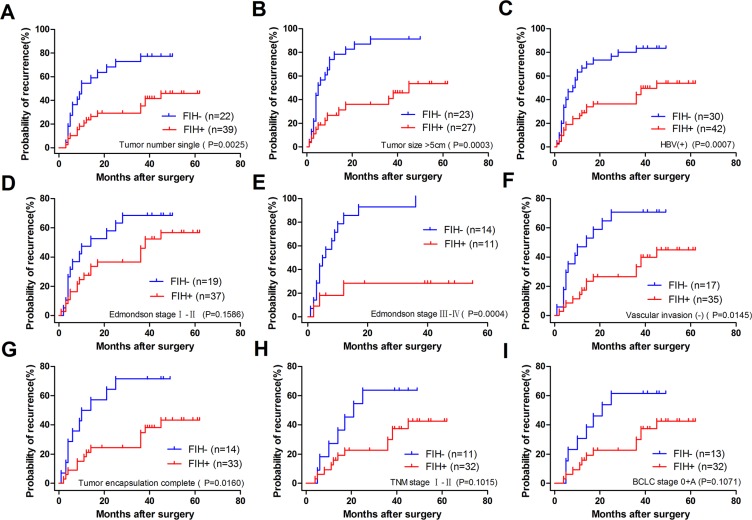
Recurrence Kaplan-Meier curves for patients with different clinicopathological characteristics according to FIH expression level in TAMs (**A**) RR of the patients with single tumor. 77.273% in FIH(–) vs. 46.038% in FIH(+), *p* = 0.0025. (**B**) RR of the patients with tumor size > 5 cm. 91.304% in FIH(–) vs. 53.489% in FIH(+), *p* = 0.0003. (**C**) RR of the patients with HBV (+). 83.333% in FIH(–) vs. 53.818% in FIH(+), *p* = 0.0007. (**D**) RR of the patients with Edmondson stage I–II. 68.421% in FIH(–) vs. 56.704 in FIH(+), *p* = 0.1586. (**E**) RR of the in patients with Edmondson stage III–IV, RR 100.000% in FIH(–) vs. 28.409% in FIH(+), *p* = 0.0004. (**F**) RR of the patients with no vascular invasion. RR 70.588% in FIH(–) vs. 44.889% in FIH(+), *p* = 0.0145. (**G**) RR of the patients with complete tumor encapsulation. RR 71.429% in FIH(–) vs. 43.273% in FIH(+), *p* = 0.0160. (**H**) RR of the patients with TNM I–II stage. RR 63.636% in FIH(–) vs. 42.550% in FIH(+), *p* = 0.1015. (**I**) RR of the patients with BCLC 0+A. RR 61.538% in FIH(–) vs. 42.550% in FIH(+), *p* = 0.1071. Log-rank test was used.

To determine how much the status of PHD3 or FIH expression contributed to the prognosis of HCC patients with different clinicopathological characteristics, we next divided the patients into several subgroups. After analysis using the stratification method, prognostic significance of PHD3 was found in HCC patients with a single tumor, tumor size > 5 cm, HBV(+), AFP ≤ 20 μg/l, Edmondson stage I–II or BCLC stage 0+A. In these clinical subgroups, patients with PHD3(–) had a significantly less favourable OS or higher RR (Figure 4). Similar results also occurred in patients with a single tumor, tumor size > 5 cm, HBV (+), no vascular invasion, complete tumor encapsulation, Edmondson stage I–II, III–IV, TNM stage I–II and BCLC stage 0+A. They had obviously reduced OS or higher RR when FIH was expressed at a lower level (Figures 5 and 6).

### Correlations among four hydroxylases, HIF-1 α, Ki-67 and MVD

Using the Spearman's rank correlation test, it was confirmed that the positive correlations among the three PHDs (PHD1 vs. PHD2, PHD2 vs. PHD3 and PHD1 vs. PHD3) were close (*r* = 0.394, 0.269 and 0.297, respectively) (Supplementary Table 4). PHD3 but not PHD1 or PHD2, had linear correlation with FIH (*r* = 0.301, *p* = 0.006).

At the same time, HIF-1α, ki-67, a commonly used tumor cell proliferation index, and MVD are biologically and clinically relevant [33]. The relevance of these mostly used histoprognostic factors [34] and the above hydroxylases was considered. Since PHDs and FIH regulate the nucleus translocation and intranuclear transcriptional activity of HIF-1 α in nuclei, respectively, HIF-1 α was evaluated only according to its staining in the nuclei. Representative images of HIF-1α, CD34 and Ki-67 staining are shown in Supplementary Figure 3. The following negative correlations were observed: PHD3 vs. Ki-67 (*r* = –0.306, *p* = 0.005), FIH vs. Ki-67 (*r* = –0.259, *p* = 0.020) and FIH vs. MVD (*r* = –0.291, *p* = 0.008). We further assessed the relationship between HIF-1 α and the above indexes, and obvious correlation (*r* = 0.245, *p* = 0.028, vs. PHD2) was found.

### Univariate and multivariate analysis of OS and DFS

Finally, univariate and multivariate analyses were performed to evaluate the potential risk factors of HCC. Univariate analysis of prognostic factors showed that PHD3 expression (*p* =0.009), FIH expression (*p* = 0.001), tumor number (*p* = 0.002), tumor encapsulation (*p* = 0.002), vascular invasion (*p <* 0.001), TNM stage (*p <* 0.001) and BCLC stage (*p <* 0.001) had significant prognostic influences on DFS (Table 2); regarding OS, FIH expression (*p <* 0.001), tumor size (*p* = 0.030), tumor number (*p* = 0.013), tumor encapsulation (*p* = 0.023), vascular invasion (*p* = 0.006), TNM stage (*p* = 0.001) and BCLC stage (*p* = 0.001) were the risk factors (Table 3). According to the results of univariate analysis of OS and DFS, we entered the significant parameters into multivariate analysis using the Cox proportional hazard model. It was confirmed that only low expression of FIH remained independent and significant unfavorable prognostic parameters for both DFS and OS (H*R* = 0.516, 95%CI 0.271–0.984, *p* = 0.044 for DFS; H*R* = 0.364, 95%CI 0.159–0.834, *p* = 0.017 for OS) from the diagnosis of HCC (Table 2 and Table 3). Other factors, like tumor number (*p* = 0.0418), tumor encapsulation (*p* = 0.325), vascular invasion (*p* = 0.792), TNM stage (*p* = 0.992) and BCLC stage (*p* = 0.159) for DFS did not reach the statistical significance (Table 2). At the same time, tumor size (*p* = 0.358), tumor number (*p* = 0.661), tumor encapsulation (*p* = 0.963), vascular invasion (*p* = 0.967), TNM stage (*p* = 0.647) and BCLC stage (*p* = 0.805) all did not reach the statistical significance for OS (Table 3). In another multivariate analysis including PHD3, none of PHD3 expression (*p* = 0.114), tumor number (*p* = 0.503), tumor encapsulation (*p* = 0.131), vascular invasion (*p* = 0.776), TNM stage (*p* = 0.697) and BCLC stage (*p* = 0.266) for DFS reached the statistical significance (Table 2). Taken together, the results indicated that the reduced expression of FIH predicts a poor prognosis and may contribute to the progression of HCC.

**Table 2:** Univariate and multivariate analysis of factors associated with disease-free survival of 81 HCC patients

Factors	Disease-free survival								
	Univariate analysis			Multivariate analysis*			Multivariate analysis^#^		
	HR	95% CI	*p*-value	HR	95% CI	*p*-value	HR	95% CI	*p*-value
Age (> 50 vs ≤ 50)	0.567	0.317–1.014	0.056						
Gender (male vs female)	0.791	0.313–1.999	0.620						
Cirrhosis (yes vs no)	1.018	0.433–2.392	0.968						
HBV (+ vs –)	2.040	0.634–6.563	0.232						
Edmondson (III–IV vs I–II)	1.587	0.879–2.865	0.126						
Serum AFP (> 20 vs ≤ 20 μg/l)	1.781	0.800–3.966	0.158						
Child-Pugh score (B vs A)	0.946	0.294–3.047	0.925						
Tumor size (> 5 vs ≤ 5 cm)	1.776	0.975–3.235	0.061						
Tumor number (multiple vs single)	2.596	1.423–4.737	**0.002**	1.329	0.577–3.061	0.503	1.416	0.610–3.286	0.418
Tumor encapsulation (none vs complete)	2.507	1.416–4.437	**0.002**	1.618	0.866–3.023	0.131	1.400	0.717–2.735	0.325
Vascular invasion (yes vs no)	2.817	1.591–4.987	**0.000**	0.859	0.303–2.441	0.776	0.869	0.305–2.475	0.792
TNM stage (III–IV vs I–II)	3.471	1.934–6.229	**0.000**	1.310	0.337–5.082	0.697	0.993	0.261–3.782	0.992
BCLC stage (B + C vs 0 + A)	3.626	2.027–6.486	**0.000**	2.304	0.529–10.039	0.266	2.804	0.667–11.782	0.159
PHD3 expression (high vs low)	0.466	0.263–0.824	**0.009**	0.615	0.336–1.124	0.114	——	——	——
FIH expression (high vs low)	0.376	0.213–0.665	**0.001**	——	——	——	0.516	0.271–0.984	**0.044**

*FIH expression was not included; #, PHD3 expression was not included; Statistically significant (*p* < 0.05).

**Table 3:** Univariate and multivariate analysis of factors associated with overall survival of 81 HCC patients

Factors	Overall survival								
	Univariate analysis			Multivariate analysis*			Multivariate analysis^#^		
	HR	95% CI	*p*-value	HR	95% CI	*p*-value	HR	95% CI	*p*-value
Age (> 50 vs ≤ 50)	0.986	0.490–1.983	0.969						
Gender (male vs female)	0.588	0.205–1.681	0.321						
Cirrhosis (yes vs no)	0.834	0.321–2.167	0.709						
HBV (+ vs –)	0.520	0.214–1.264	0.149						
Edmondson (III–IV vs I–II)	1.586	0.774–3.248	0.208						
Serum AFP (> 20 vs ≤ 20 μg/l)	1.545	0.595–4.013	0.372						
Child-Pugh score (B vs A)	0.551	0.075–4.035	0.557						
Tumor size (> 5 vs ≤ 5cm)	2.429	1.090–5.417	**0.030**				1.532	0.616–3.809	0.358
Tumor number (multiple vs single)	2.505	1.217–5.155	**0.013**				1.243	0.470–3.284	0.661
Tumor encapsulation (none vs complete)	2.255	1.119–4.544	**0.023**				1.020	0.436–2.385	0.963
Vascular invasion (yes vs no)	2.647	1.319–5.311	**0.006**				0.974	0.279–3.397	0.967
TNM stage (III–IV vs I–II)	3.603	1.700–7.635	**0.001**				1.564	0.230–10.638	0.647
BCLC stage (B + C vs 0 + A)	3.358	1.614–6.988	**0.001**				1.294	0.168–9.997	0.805
PDH3 expression (high vs low)	0.534	0.266–1.072	0.078				——	——	——
FIH expression (high vs low)	0.261	0.124–0.547	**0.000**				0.364	0.159–0.834	**0.017**

*FIH expression was not included. #, PHD3 expression was not included; Statistically significant (*p* < 0.05).

## DISCUSSION

Although different PHD displays its own tissue and cell specific expression pattern as well as particular subcellular distribution [29], positive correlations were found between three PHD isoforms in our study, and more interestingly, PHD3 also had linear correlation with FIH in HCC patients. Similar correlation was also observed in patients with non-small cell lung cancer (NSCLC), pancreatic endocrine tumors and head and neck squamous cell carcinoma (HNSCC) [34–36]. An important fact should be noticed that PHDs not only served as the regulator of HIF, they could also be the transcriptional targets of HIF. At the same time, it is widely accepted that under normoxia, PHDs and FIH act synergistically to restrict the activity of HIF to minimum. Obviously, studying these hydroxylases together in a certain type of tumor will be more beneficial than study them separately. To our knowledge, it is for the first time that such clinical investigations on the correlation between four HIF hydroxylases in HCC had been conducted. This provoked us to make a thorough comparison between their expression pattern in HCC and normal tissue.

Other than altered expression of PHD1 and PHD2 in NSCLC, pancreaticobiliary cancer, cervical carcinoma and CRC [27, 37–40], in current study, PHD1 and PHD2 remained unchanged in HCC compared with normal liver tissue, although PHD2 was considered as most abundant and important PHD isoform in setting the steady-state level of HIF-1α [41]. Our results suggested that neither PHD1 nor PHD2 was involved in the progression of HCC. As pointed out by Peurala, the intracellular shuffling of PHD2 is a cancer-type-specific phenomenon [42]. Although, different with our results, Li Zhen et al. reported that higher PHD2 expression was prevalent in a study on 20 pairs of HCC tumor and matched normal tissue [43], this result was not further verified by western blotting and real-time PCR. Meanwhile, the small sample size in this study is a weakness with regard to conducting statistical analysis.

At the same time, data from the present study confirmed a lower expression of PHD3 and FIH in tumor tissue, which is also correlated with more aggressive behaviors of HCC. Similarly, Tanaka and his colleagues found that PHD3 was weakly or even negatively stained in HCC [31]. This result suggests the possibility of PHD3 and FIH-1 as suppressor in HCC tumorigenesis. Unlike most of the studies targeting FIH describing a commonly reduced expression in tumor [44–46], clinical investigations on PHD3 had controversial results, such as elevated in pancreaticobiliary cancer and HNSCC [39, 47] but reduced in gastric cancer [48]. To our opinion, this diversity of PHD3 expression might be at least partly due to the tissue heterogeneity. It is also worth mention that several independent clinical trials on CRC not only indicated a decreased expression of PHD3 and FIH, but also revealed their association with higher tumor grade and metastasis [27, 45, 46, 49, 50].

What interests us most is that the expression of FIH was obviously decreased in patients with more aggressive HCC, including the clinical features like vascular invasion, no complete tumor encapsulation or higher stage. This is in accordance with what others found in CRC and invasive breast cancer [32, 46], additionally, higher expression of FIH related to a low incidence of metastasis to the lymph nodes in NSCLC [35]. In additional to the conventional hypoxia dependent FIH inside tumor, hypoxia independent pathway might also take part in the regulatory effect of FIH in cellular migration, for example, it was also confirmed to be the target of miR-31 and miR-135b in tumorigenesis [44, 51]. Similarly, reduction of PHD3 expression was more obviously in the HCC patients with higher tumor size or less complete tumor encapsulations. Combining with the results from CRC and gastric cancer [48, 49], we have reason to assume that the inhibition of PHD3 in HCC might rely on some growth- or metastasis-promoting factors. Several studies further provided compelling evidences to elucidate the effective of PHD3 in tumorigenesis and metastasis. It could inhibit the tumor migratory potential both *in vivo* and *in vitro* by reducing matrix metalloproteinases production, blocking the colony formation, decrease the mitochondrial ATP generation, suppress the beta-catenin/T-cell factor signaling and inhibit IKKβ/NF-κB signaling, independent of its hydroxylase activity [48–50]. On the other hand, activity of PHD3 to induce apoptosis through HIF-1-dependent, or independent pathway by activation of caspase-3 also contributed to the above correlation [52, 53]. More interestingly, when comparison was carried out between multiple groups, patients with lower expression of both PHD3 and FIH exhibited the least chance of forming tumor encapsulation and highest TNM stage. It was found in HeLa cells, when cells were combined silenced of PHD2 together with PHD3, up-regulation of HIF-1α was more obvious than silencing PHD2 or PHD3 separately [54, 55]. Simultaneously inhibition of PHD and FIH could enhance the activity more seriously than inhibition of two PHD alone [56]. We speculate that synergistic enhancing both the stabilization and transcriptional activity of HIF-1α by combined low expression of PHD3 and FIH is the reason for the worse clinical outcome. Even through the complete metastasis-promoting event involving the combined lower expression of FIH and PHD3 still need further exploration, our result is still of high value to provide the first evidence that lower level of PHD3 and FIH may be an important co-regulator of metastasis in HCC.

In view of the fact that, as the target of PHD3 and FIH, HIF is not only a marker of poor prognosis in HCC [57], but also an important regulator of uncontrolled cell proliferation and neovascularization in tumor [58]. In order to explore the possible mechanism that how PHD3 or FIH function as tumor suppressor, HIF-1α, Ki-67 and MVD were considered. It was beyond our expectations that, no correlation between PHD3 and HIF-1α was found in current study. It was similar with what other groups found in colorectal cancer and NSLC [35, 37]. Study from Appelhof and his colleagues might help us explain this observation. Using small interfering RNA-mediated suppression of PHDs model, they confirmed a stronger inhibitory effects of PHD3 on HIF-2a than HIF-1a [23]. And, we should not neglect that HIF-1-dependent PHD3 induction forms an auto-regulatory loop controlling HIF-1α [59–61]. That is to say nuclear accumulation of HIF-1α, caused by lower level of PHD3, could also up regulate the expression of PHD3. This could explain why PHD3 had no obvious negative correlation with nuclear HIF-1 α in our study.

However, PHD2 was positively correlated with the nuclear staining of HIF-1α in HCC. Similar phenomena were also reported in NSCLC and HNSCC [35, 36]. It might be because of PHD2 as a physiological rather than a pathological regulator of HIF. Because there are several other oxygen-independent pathways involving in the regulation of HIF-1α [17], it could be understandable that in HCC, PHD2 alone might not be sufficient to decrease the accumulation of HIF-1 or form a negative correlation. It is no doubt that the regulation of HIF is far more complicated than we expected in HCC.

Although FIH did not show close correlation with the nuclear staining of HIF-1α, since it primarily modulates the activity rather than expression or degradation of the latter, we still found that the patients with lower expression of FIH had much higher MVD, same as in HNSCC [51]. As one of the hallmark of tumor, neovascularization is a well-known downstream event of activated HIF-1a through up-regulation of erythropoietin, vascular endothelial growth factor and its receptor [15]. It is easily to understand why there was a reverse association between FIH and vascular generation in HCC, which was also proved in Hep3B cells *in vitro* and HNSCC [44, 62].

As for the other histopathological factor, also a commonly used tumor cell proliferation index, Ki-67 showed strong negative correlation with both PHD3 and FIH. Reduced expression of FIH caused a relatively higher transcriptional ability of HIF-1α and in turn, higher expression of its targets genes which including several pro-proliferation factors like glucose transporter 1 [46]. FIH knocking out experiments confirmed enhanced proliferation both *in vitro* and *in vivo* [44–46]. Compared with FIH, this negative influence of PHD3 on proliferation was more serious in our study, and also confirmed in several other tumor types, including CRC, gastric cancer and breast cancer, which might involving HIF-1-dependent or independent pathway, such as IKKβ/NF-κB, the beta-catenin/T-cell factor, activation of caspase-3 and phosphorylation of focal adhesion kinase [42, 48, 49, 53]. Another two studies provided further information of human hepatoma cell line. By using PHD3 steady expression plasmid transfected HepG2 and constructing HepG2-bearing subcutaneous tumor in nude mice, researchers found out that PHD3 gene may inhibit proliferation and induce apoptosis by activating caspase-3 activity [63, 64]. Taken together, the tumor suppressive effect of FIH and PHD3 should exert through inhibiting proliferation or blocking neovascularization.

Despite both PHD1 and PHD2 had no obvious change between tumor and normal tissue; further statistic investigation presented a reverse correlation between the tumor PHD1 level and recurrence of HCC patients (Seen in Supplementary Figure 4). We also found that patients with low level of PHD1 tend to have incomplete tumor encapsulation, however, patients with higher PHD2 level are more often to have higher serum AFP (Supplementary Table 5). In regard to PHD2, different conclusions had been reached dependent on the cancer type, some indicating PHD2 as either a poor or favorable prognostic factor [37, 43], however, some similar to ours indicating no prognostic significance of PHD2 [39]. We hold the opinion that it is still too early to make the final judgment about the role of PHD2 in HCC tumorigenesis. As expected, lower expression of PHD3 or FIH correlated with a poor prognosis of HCC, including the higher RR, shorter DFS or shorter OS, especially with more serious early stage of recurrence. As we know, HCC recurrence, generally in the hepatic remnant, occurs in very high percentage of cases after resective surgery [65]. Identification of poor prognostic factors in early recurrence, not only prompt us to perform enhanced surveillance for recurrence following surgical resection, but also guide the doctors in the choice of therapy for individuals.

There is a tendency, quite supportive to our finding, that the higher expression of FIH, the longer survival time or lower risk of recurrence those patients will have [28, 46]. In current study, multivariable analysis further identified the reduced expression of FIH as an independent and significant prognostic parameter for both DFS and OS. This was also confirmed in CRC and ccRCC [28, 46]. We are quite impressed with the phenomena that even within the nucleus, unhydroxylated HIF-1α still could be asparaginyl-hydroxylated by nuclear FIH, as a compensation for the function of cytoplasm PHDs and FIH [56]. That is to say, for a maximum efficiency, both cytoplasmic and nuclear FIH should work simultaneously. For this reason, we think it maybe not comprehensive to consider cytoplasmic FIH-1 as an independent poor prognostic factor based on the statistics only focusing on the cytoplasm FIH [32], especially when as high as 62.71% nucleus expression of FIH was detected in invasive breast cancer cells. In our opinion, if FIH in both cytoplasm and nucleus had be taken into consideration together, the results in this study would be more beneficial [32]. Actually, prognostic significance was more obvious when nuclear FIH was considered alone [66].

Much more important finding is obtained from multigroup comparison. Patients with simultaneous underexpression of FIH and PHD3 not only exhibited the least chance of forming tumor encapsulation and highest TNM stage, but also had lowest OS and highest recurrent rate. Our study raised the possibility that combining FIH and PHD3 as co-factors will be more accurate to predict the outcome of HCC patients. It could not be ignored that the present study was a single-institute research with small sample size. The inherent feature of biomarkers verifying, and the nature of retrospective study, are also inevitable limitations. A more rigorous evaluation and validation of PHD3 and FIH in multi-institute, large-scale prospective trials performance by multi-institute are advocated. We further compared the cellular expression of PHD3 and FIH in 2 normal liver cell line and 12 HCC cell line. It was (results were shown in Supplementary Figure 5), hoping it would be beneficial for the further investigation on detailed mechanisms.

Taken together, we confirmed a reduced expression of PHD3 and FIH in HCC which is associated with more aggressive behaviors. Combining with the fact that the hydroxylase activity of PHD3 and FIH is partially decreased under the relatively hypoxic condition in HCC, the action of lower level of PHD3 and FIH, individually and collectively, as unfavorable prognostic factors for HCC has much higher value. These data suggest that PHD3 and FIH are potential therapeutic targets for HCC treatment.

## MATERIALS AND METHODS

### Patients and tumor samples

Eighty-one pairs of tumors and adjacent non-tumor liver tissues (ANLTs) were collected from patients with HCC who had undergone surgical resection at the Hepatic Surgery Center, Tongji Hospital of Huazhong University of Science and Technology, Wuhan, China, between July 2010 and June 2013. All of the recruited patients in this study were not subjected to preoperative radiotherapy and/or chemotherapy. The HCC diagnosis was based on the histochemistry assay according to World Health Organization criteria [67, 68]. The recurrence of intrahepatic tumor was confirmed with imaging and the elevation of tumor markers and/or histology. Whole-body positron emission tomography/computed tomography was used to assess extra-hepatic metastasis. All of the clinical data, including the patient characteristics, clinical presentation, tumor differentiation, lesion sites and laboratory findings, were collected from the hospital information system. OS was defined as the interval between the date of resection and date of death or last follow-up. The recurrence time was calculated from the date of operation to the date of diagnosis of intrahepatic tumor or metastasis in other organs. Informed content was obtained, and access to human samples was carried out in accordance with the approved consent of the Ethics Committee of Tongji Hospital (Approval ID: TJ-C20141113).

### Cell lines and cell culture

The cell line from noncancerous liver tissue QSG-7701 and HCC cell line PLC/PRF-5 were all purchased from cell bank of Chinese Academy of Sciences (Shanghai, China). Human fetal liver cell line HL-7702, HCC cell line HepG2, Hep3B, Huh7, SK-Hep1, SMMC7721 and Bel7402 were obtained from China Center for Type Culture Collection (CCTCC, Wuhan, China). Human HCC cell lines MHCC97-L, MHCC97-H and HCCLM3 were purchased from Liver Cancer Institute, Zhongshan Hospital, Fudan University, Shanghai, China [69]. HLE and HLF cells were kindly provided by Shanshan Wang and Gang Li (Department of Molecular Biology, Peking University Health Science Center, Beijing, China) [70]. These cell lines were cultured in Dulbecco's modified Eagle's medium (Invitrogen) supplemented with 10% fetal bovine serum (Gibco, Grand Island, NY) ,100 U/ml penicillin and 100 μg/ml streptomycin and maintained at 37°C in a 5% CO_2_, 95% humidified atmosphere incubator.

### TMA construction and IHC staining

As previously reported [71], the TMAs were constructed using a tissue array instrument (Quick-Ray, UT06; UNITMA, Korea). Briefly, tissue cores (2 mm in diameter) were punched from representative formalin-fixed paraffin-embedded tissue and put in order in the TMA blocks, which were then sectioned into series of 4-μm-thick slices. The expression of target proteins in TMA was tested through standard IHC methods, which were performed using primary antibodies, including mouse anti-PHD1 (1:100; Abcam, Cambridge, UK), rabbit anti-PHD2 (1:100; NOVUS Biologicals, Littleton CO, USA), rabbit anti-PHD3 (1:250; NOVUS Biologicals, Littleton CO, USA), mouse anti-FIH (1:200; NOVUS Biologicals, Littleton CO, USA), mouse anti-HIF-1α (1:400; Abcam, Cambridge, UK), mouse anti-Ki-67 (1:200; Dako, Glostrup, Denmark) and rabbit anti-CD34 (1:350; Abcam, Cambridge, UK), incubated overnight at 4°C, and then incubated with HRP-conjugated secondary antibodies (1:5000; Envision detection kit, DAKO) for 30 min at room temperature.

### Assessment of four hydroxylases

To score the immunostaining of four hydroxylases as we published previously [34, 72], the intensity of IHC was classified into 4 categories—0, 1, 2 and 3—corresponding to no staining, weak staining, moderate staining and strong staining, respectively. The percentage of positively staining tumor cells was classified into 5 categories—0, 1, 2, 3, 4—which corresponding to < 10%, 10–25%, 26–50%, 51–75% and > 75%, respectively. The product of the staining intensity score and percentage of positive cells was considered the final score of target protein expression, which ranged from 0 (no staining) to 12 (75–100% of cells with 3 staining intensity scores). TAM tissue with the final score ≥ 6 was considered as higher expression or (+), while < 6 was considered as lower expression or (–). All the immunostaining results were assessed and scored independently by two pathologists.

### Evaluation of HIF-1α

HIF-1α was scored according to the presence of nuclear staining as previously published [73]. Only cells with completely and darkly stained nuclei were interpreted as positive expression. Additionally, because of the narrow range of the staining intensity, HIF-1α was only scored as 1+ and 0 according to the presence and absence of nuclear expression, respectively.

### Evaluation of MVD

MVD in tumor tissue was determined by the presence of CD34 (as an endothelial marker) [74]. Briefly, tumor TAMs from HCC patients were scanned at low-power fields (×40) to find the areas that showed the most intense neovascularization (hot spots). Individual microvessels were counted in three fields at high power fields (HPF) (×200). Any positively stained endothelial cells or endothelial cell cluster that was clearly separated from adjacent microvessels, tumor cells, and connective elements was considered a single and countable microvessel. The final MVD was the mean value obtained from the counts of three fields, expresses as the absolute number of microvessels/HPF.

### Evaluation of the Ki-67 labeling index

Five representative areas of each section were chosen to count the immunoreactive cells, and at least 200 cells from each area were evaluated (×400). The percentage of Ki-67-positive nuclei to the total number of nuclei was used as the labeling index [75].

### Western blot analysis

Proteins in the tumor and paired ANLTs in a set of 24 randomly selected cases (from 81 HCC patients) were extracted using RIPA lysis buffer (50 mM Tris at pH 7.4, 150 mM NaCl, 1% NP-40, 0.5% sodium deoxycholate, and 0.1% SDS) with phenylmethanesulphonyl fluoride (17.4 μg/μl) and a protease inhibitor cocktail (0.89 μg/μl, Sigma-Aldrich). After the protein concentration was determined using a BCA Protein Assay Kit (Pierce), the proteins were separated on SDS-polyacrylamide gels followed by transfer to polyvinylidene difluoride membranes, which were blocked in 5% fat-free milk with TBS-Tween-20 at room temperature for 1 h, followed by incubation with primary antibodies mouse anti-PHD1 (1:1000; Abcam, Cambridge, UK), rabbit anti-PHD2 (1:1000; NOVUS Biologicals, Littleton CO, USA), rabbit anti-PHD3 (1:1000; NOVUS Biologicals, Littleton CO, USA), mouse anti-FIH (1:200; NOVUS Biologicals, Littleton CO, USA), or anti-β actin antibody (1:1000; Santa Cruz, USA) at 4°C overnight. Following incubation with an HRP-conjugated secondary antibody (1:5000; Jackson Immmuno Research Laboratories, INC, West Grove, PA, USA), the blots were visualized using enhanced chemiluminescence (Pierce Biotechnology, USA) and were exposed to the Syngene GBOX/iCHE gel imaging systems. The intensity of each band was analyzed by Image J and normalized to β-actin.

### Real-time PCR

The tumor and paired ANLTs in a set of 40 randomly selected cases (from 81 HCC patients) were suspended in Trizol (Invitrogen, Carlsbad, CA, USA), and total RNA was extracted and reverse transcribed according to the manufacturer's instructions (Revertra Ace-a kit; TOYOBO, Osaka, Japan). Real-time PCR of human PHD1, PHD2, PHD3, FIH and GAPDH was performed using a Bio-Rad CFX Connect Real-time PCR Detection system (Bio-Rad Laboratories, Richmond, CA, USA) according to the manufacturer's instructions for the SYBR Green Master mix (TOYOBO, Osaka, Japan). GAPDH was served as the internal control. The forward and reverse primers are shown in Supplementary Table 6. The relative expression of the target gene was calculated using the comparative CT method. In details, ΔCt(A) = Ct value of the target gene –Ct value of GAPDH in ANLTs. ΔCt(T) = Ct value of the target gene –Ct value of GAPDH in HCC tumor tissue. The target mRNA of the tumor is 2^−ΔΔCt^ -fold of that in paired ANLTs (ΔΔCt = ΔCt(T) –ΔCt(A)). ΔΔCt ≥ 1 (or –ΔΔCt ≤ –1) indicates that the expression of the target gene is decreased in tumors. ΔΔCt ≤ –1 (or –ΔΔCt ≥ 1) indicates that the expression of the target gene is increased in tumors.

### Statistical methods

All of the data were analyzed by SPSS version 17.0 or GraphPad Prism version 5.0. The IHC score of the target protein in TAMs was compared using the non-parametric approach (Wilcoxon signed-rank test) between the tumor tissue and paired ANLTs. The relative protein level in the western blotting assay and mRNA level in real-time PCR were compared with paired *t* test. Pearson χ^2^ test and Fisher exact tests were applied to analyze the difference between categorical variables, as well as the correlation between PHD3 or FIH expression and clinicopathological features. If multiple comparisons were further used, pairwise χ^2^ test was applied with the Bonferroni correction. Spearman rank correlation was applied to assess the correlation among the four hydroxylases, HIF-1α, MVD and Ki-67. OS or recurrence curves were plotted using the Kaplan-Meier method and were evaluated for the statistical significance using the log-rank test. Variables with significant results in univariate analysis were entered into the multivariate Cox proportional hazards model. *P <* 0.05 was considered to indicate a statistically significant difference.

## SUPPLEMENTARY MATERIALS TABLES AND FIGURES



## References

[1] Zhu AX (2006). Systemic therapy of advanced hepatocellular carcinoma: how hopeful should we be?. The oncologist.

[2] Forner A, Llovet JM, Bruix J (2012). Hepatocellular carcinoma. Lancet.

[3] Jemal A, Bray F, Center MM, Ferlay J, Ward E, Forman D (2011). Global cancer statistics. CA Cancer J Clin.

[4] Ye SL, Chen X, Yang J, Bie P, Zhang S, Liu F, Liu L, Zhou J, Dou K, Hao C, Shao G, Xia Q, Chen Y (2016). Safety and efficacy of sorafenib therapy in patients with hepatocellular carcinoma: final outcome from the Chinese patient subset of the GIDEON study. Oncotarget.

[5] Marrero JA, Kudo M, Venook AP, Ye SL, Bronowicki JP, Chen XP, Dagher L, Furuse J, Geschwind JH, de Guevara LL, Papandreou C, Takayama T, Sanyal AJ (2016). Observational registry of sorafenib use in clinical practice across Child-Pugh subgroups: The GIDEON study. Journal of hepatology.

[6] Xiao H, Zhang B, Mei B, Zuo C, Wei G, Wang R, Zhang B, Chen X (2015). Hepatic resection for hepatocellular carcinoma in patients with portal hypertension: a long-term benefit compared with transarterial chemoembolization and thermal ablation. Medicine.

[7] Wang Q, Lau WY, Zhang B, Zhang Z, Huang Z, Luo H, Chen X (2014). Preoperative total cholesterol predicts postoperative outcomes after partial hepatectomy in patients with chronic hepatitis B- or C-related hepatocellular carcinoma. Surgery.

[8] Zhu P, Lau WY, Chen YF, Zhang BX, Huang ZY, Zhang ZW, Zhang W, Dou L, Chen XP (2012). Randomized clinical trial comparing infrahepatic inferior vena cava clamping with low central venous pressure in complex liver resections involving the Pringle manoeuvre. The British journal of surgery.

[9] Zhang B, Dong W, Luo H, Zhu X, Chen L, Li C, Zhu P, Zhang W, Xiang S, Zhang W, Huang Z, Chen XP (2016). Surgical treatment of hepato-pancreato-biliary disease in China: the Tongji experience. Science China Life sciences.

[10] Kaseb AO, Hassan M, Lacin S, Abdel-Wahab R, Amin HM, Shalaby A, Wolff RA, Yao J, Rashid A, Vennapusa B, Feng J, Ohtomo T (2016). Evaluating clinical and prognostic implications of Glypican-3 in hepatocellular carcinoma. Oncotarget.

[11] Cai Y, Zhang J, Wu J, Li ZY (2015). Oxygen transport in a three-dimensional microvascular network incorporated with early tumour growth and preexisting vessel cooption: numerical simulation study. BioMed research international.

[12] Wijffels KI, Marres HA, Peters JP, Rijken PF, van der Kogel AJ, Kaanders JH (2008). Tumour cell proliferation under hypoxic conditions in human head and neck squamous cell carcinomas. Oral oncology.

[13] Deynoux M, Sunter N, Herault O, Mazurier F (2016). Hypoxia and Hypoxia-Inducible Factors in Leukemias. Frontiers in oncology.

[14] Rankin EB, Giaccia AJ (2016). Hypoxic control of metastasis. Science.

[15] Ruan K, Song G, Ouyang G (2009). Role of hypoxia in the hallmarks of human cancer. Journal of cellular biochemistry.

[16] Folkman J (1971). Tumor angiogenesis: therapeutic implications. The New England journal of medicine.

[17] Masoud GN, Li W (2015). HIF-1alpha pathway: role, regulation and intervention for cancer therapy. Acta pharmaceutica Sinica B.

[18] Wigerup C, Pahlman S, Bexell D (2016). Therapeutic targeting of hypoxia and hypoxia-inducible factors in cancer. Pharmacology & therapeutics.

[19] Semenza GL (2010). Defining the role of hypoxia-inducible factor 1 in cancer biology and therapeutics. Oncogene.

[20] Chen Y, Hao H, He S, Cai L, Li Y, Hu S, Ye D, Hoidal J, Wu P, Chen X (2010). Lipoxin A4 and its analogue suppress the tumor growth of transplanted H22 in mice: the role of antiangiogenesis. Mol Cancer Ther.

[21] Liu W, Shen SM, Zhao XY, Chen GQ (2012). Targeted genes and interacting proteins of hypoxia inducible factor-1. International journal of biochemistry and molecular biology.

[22] Epstein AC, Gleadle JM, McNeill LA, Hewitson KS, O'Rourke J, Mole DR, Mukherji M, Metzen E, Wilson MI, Dhanda A, Tian YM, Masson N, Hamilton DL (2001). C. elegans EGL-9 and mammalian homologs define a family of dioxygenases that regulate HIF by prolyl hydroxylation. Cell.

[23] Appelhoff RJ, Tian YM, Raval RR, Turley H, Harris AL, Pugh CW, Ratcliffe PJ, Gleadle JM (2004). Differential function of the prolyl hydroxylases PHD1, PHD2, and PHD3 in the regulation of hypoxia-inducible factor. J Biol Chem.

[24] Cavadas MA, Nguyen LK, Cheong A (2013). Hypoxia-inducible factor (HIF) network: insights from mathematical models. Cell communication and signaling.

[25] Li Y, Zhang D, Wang X, Yao X, Ye C, Zhang S, Wang H, Chang C, Xia H, Wang YC, Fang J, Yan J, Ying H (2015). Hypoxia-inducible miR-182 enhances HIF1alpha signaling via targeting PHD2 and FIH1 in prostate cancer. Scientific reports.

[26] Madsen CD, Pedersen JT, Venning FA, Singh LB, Moeendarbary E, Charras G, Cox TR, Sahai E, Erler JT (2015). Hypoxia and loss of PHD2 inactivate stromal fibroblasts to decrease tumour stiffness and metastasis. EMBO reports.

[27] Rawluszko AA, Bujnicka KE, Horbacka K, Krokowicz P, Jagodzinski PP (2013). Expression and DNA methylation levels of prolyl hydroxylases PHD1, PHD2, PHD3 and asparaginyl hydroxylase FIH in colorectal cancer. BMC cancer.

[28] Kroeze SG, Vermaat JS, van Brussel A, van Melick HH, Voest EE, Jonges TG, van Diest PJ, Hinrichs J, Bosch JL, Jans JJ (2010). Expression of nuclear FIH independently predicts overall survival of clear cell renal cell carcinoma patients. European journal of cancer.

[29] Metzen E, Berchner-Pfannschmidt U, Stengel P, Marxsen JH, Stolze I, Klinger M, Huang WQ, Wotzlaw C, Hellwig-Burgel T, Jelkmann W, Acker H, Fandrey J (2003). Intracellular localisation of human HIF-1 alpha hydroxylases: implications for oxygen sensing. Journal of cell science.

[30] Soilleux EJ, Turley H, Tian YM, Pugh CW, Gatter KC, Harris AL (2005). Use of novel monoclonal antibodies to determine the expression and distribution of the hypoxia regulatory factors PHD-1, PHD-2, PHD-3 and FIH in normal and neoplastic human tissues. Histopathology.

[31] Tanaka T, Li TS, Urata Y, Goto S, Ono Y, Kawakatsu M, Matsushima H, Hirabaru M, Adachi T, Kitasato A, Takatsuki M, Kuroki T, Eguchi S (2015). Increased expression of PHD3 represses the HIF-1 signaling pathway and contributes to poor neovascularization in pancreatic ductal adenocarcinoma. Journal of gastroenterology.

[32] Tan EY, Campo L, Han C, Turley H, Pezzella F, Gatter KC, Harris AL, Fox SB (2007). Cytoplasmic location of factor-inhibiting hypoxia-inducible factor is associated with an enhanced hypoxic response and a shorter survival in invasive breast cancer. Breast cancer research.

[33] Berghoff AS, Ilhan-Mutlu A, Wohrer A, Hackl M, Widhalm G, Hainfellner JA, Dieckmann K, Melchardt T, Dome B, Heinzl H, Birner P, Preusser M (2014). Prognostic significance of Ki67 proliferation index, HIF1 alpha index and microvascular density in patients with non-small cell lung cancer brain metastases. Strahlentherapie und Onkologie.

[34] Couvelard A, Deschamps L, Rebours V, Sauvanet A, Gatter K, Pezzella F, Ruszniewski P, Bedossa P (2008). Overexpression of the oxygen sensors PHD-1, PHD-2, PHD-3, and FIH Is associated with tumor aggressiveness in pancreatic endocrine tumors. Clin Cancer Res.

[35] Giatromanolaki A, Koukourakis MI, Pezzella F, Turley H, Sivridis E, Bouros D, Bougioukas G, Harris AL, Gatter KC (2008). Expression of prolyl-hydroxylases PHD-1, 2 and 3 and of the asparagine hydroxylase FIH in non-small cell lung cancer relates to an activated HIF pathway. Cancer Lett.

[36] Jokilehto T, Rantanen K, Luukkaa M, Heikkinen P, Grenman R, Minn H, Kronqvist P, Jaakkola PM (2006). Overexpression and nuclear translocation of hypoxia-inducible factor prolyl hydroxylase PHD2 in head and neck squamous cell carcinoma is associated with tumor aggressiveness. Clinical cancer research.

[37] Xie G, Zheng L, Ou J, Huang H, He J, Li J, Pan F, Liang H (2012). Low expression of prolyl hydroxylase 2 is associated with tumor grade and poor prognosis in patients with colorectal cancer. Exp Biol Med (Maywood).

[38] Andersen S, Donnem T, Stenvold H, Al-Saad S, Al-Shibli K, Busund LT, Bremnes RM (2011). Overexpression of the HIF hydroxylases PHD1, PHD2, PHD3 and FIH are individually and collectively unfavorable prognosticators for NSCLC survival. PloS one.

[39] Gossage L, Zaitoun A, Fareed KR, Turley H, Aloysius M, Lobo DN, Harris AL, Madhusudan S (2010). Expression of key hypoxia sensing prolyl-hydroxylases PHD1, -2 and -3 in pancreaticobiliary cancer. Histopathology.

[40] Roszak A, Kedzia W, Malkowska-Walczak B, Pawlik P, Kedzia H, Luczak M, Lianeri M, Jagodzinski PP (2011). Reduced expression of PHD2 prolyl hydroxylase gene in primary advanced uterine cervical carcinoma. Biomedicine & pharmacotherapy.

[41] Hewitson KS, Schofield CJ, Ratcliffe PJ (2007). Hypoxia-inducible factor prolyl-hydroxylase: purification and assays of PHD2. Methods in enzymology.

[42] Peurala E, Koivunen P, Bloigu R, Haapasaari KM, Jukkola-Vuorinen A (2012). Expressions of individual PHDs associate with good prognostic factors and increased proliferation in breast cancer patients. Breast cancer research and treatment.

[43] Zhen L, Shijie N, Shuijun Z (2014). Tumor PHD2 expression is correlated with clinical features and prognosis of patients with HCC receiving liver resection. Medicine.

[44] Liu CJ, Tsai MM, Hung PS, Kao SY, Liu TY, Wu KJ, Chiou SH, Lin SC, Chang KW (2010). miR-31 ablates expression of the HIF regulatory factor FIH to activate the HIF pathway in head and neck carcinoma. Cancer research.

[45] Chen T, Yao LQ, Shi Q, Ren Z, Ye LC, Xu JM, Zhou PH, Zhong YS (2014). MicroRNA-31 contributes to colorectal cancer development by targeting factor inhibiting HIF-1alpha (FIH-1). Cancer biology & therapy.

[46] Chen T, Ren Z, Ye LC, Zhou PH, Xu JM, Shi Q, Yao LQ, Zhong YS (2015). Factor inhibiting HIF1alpha (FIH-1) functions as a tumor suppressor in human colorectal cancer by repressing HIF1alpha pathway. Cancer biology & therapy.

[47] Hogel H, Rantanen K, Jokilehto T, Grenman R, Jaakkola PM (2011). Prolyl hydroxylase PHD3 enhances the hypoxic survival and G1 to S transition of carcinoma cells. PloS one.

[48] Cui L, Qu J, Dang S, Mao Z, Wang X, Fan X, Sun K, Zhang J (2014). Prolyl hydroxylase 3 inhibited the tumorigenecity of gastric cancer cells. Molecular carcinogenesis.

[49] Xue J, Li X, Jiao S, Wei Y, Wu G, Fang J (2010). Prolyl hydroxylase-3 is down-regulated in colorectal cancer cells and inhibits IKKbeta independent of hydroxylase activity. Gastroenterology.

[50] Radhakrishnan P, Ruh N, Harnoss JM, Kiss J, Mollenhauer M, Scherr AL, Platzer LK, Schmidt T, Podar K, Opferman JT, Weitz J, Schulze-Bergkamen H, Koehler BC (2016). Prolyl Hydroxylase 3 Attenuates MCL-1-Mediated ATP Production to Suppress the Metastatic Potential of Colorectal Cancer Cells. Cancer research.

[51] Zhang L, Sun ZJ, Bian Y, Kulkarni AB (2013). MicroRNA-135b acts as a tumor promoter by targeting the hypoxia-inducible factor pathway in genetically defined mouse model of head and neck squamous cell carcinoma. Cancer letters.

[52] Schlisio S, Kenchappa RS, Vredeveld LC, George RE, Stewart R, Greulich H, Shahriari K, Nguyen NV, Pigny P, Dahia PL, Pomeroy SL, Maris JM, Look AT (2008). The kinesin KIF1Bbeta acts downstream from EglN3 to induce apoptosis and is a potential 1p36 tumor suppressor. Genes & development.

[53] Su Y, Loos M, Giese N, Hines OJ, Diebold I, Gorlach A, Metzen E, Pastorekova S, Friess H, Buchler P (2010). PHD3 regulates differentiation, tumour growth and angiogenesis in pancreatic cancer. British journal of cancer.

[54] Stiehl DP, Wirthner R, Koditz J, Spielmann P, Camenisch G, Wenger RH (2006). Increased prolyl 4-hydroxylase domain proteins compensate for decreased oxygen levels. Evidence for an autoregulatory oxygen-sensing system. J Biol Chem.

[55] Koditz J, Nesper J, Wottawa M, Stiehl DP, Camenisch G, Franke C, Myllyharju J, Wenger RH, Katschinski DM (2007). Oxygen-dependent ATF-4 stability is mediated by the PHD3 oxygen sensor. Blood.

[56] Nguyen LK, Cavadas MA, Scholz CC, Fitzpatrick SF, Bruning U, Cummins EP, Tambuwala MM, Manresa MC, Kholodenko BN, Taylor CT, Cheong A (2013). A dynamic model of the hypoxia-inducible factor 1alpha (HIF-1alpha) network. J Cell Sci.

[57] Bangoura G, Liu ZS, Qian Q, Jiang CQ, Yang GF, Jing S (2007). Prognostic significance of HIF-2alpha/EPAS1 expression in hepatocellular carcinoma. World journal of gastroenterology.

[58] Li S, Yao D, Wang L, Wu W, Qiu L, Yao M, Yao N, Zhang H, Yu D, Ni Q (2011). Expression characteristics of hypoxia-inducible factor-1alpha and its clinical values in diagnosis and prognosis of hepatocellular carcinoma. Hepatitis monthly.

[59] Aprelikova O, Chandramouli GV, Wood M, Vasselli JR, Riss J, Maranchie JK, Linehan WM, Barrett JC (2004). Regulation of HIF prolyl hydroxylases by hypoxia-inducible factors. Journal of cellular biochemistry.

[60] Berchner-Pfannschmidt U, Yamac H, Trinidad B, Fandrey J (2007). Nitric oxide modulates oxygen sensing by hypoxia-inducible factor 1-dependent induction of prolyl hydroxylase 2. The Journal of biological chemistry.

[61] Marxsen JH, Stengel P, Doege K, Heikkinen P, Jokilehto T, Wagner T, Jelkmann W, Jaakkola P, Metzen E (2004). Hypoxia-inducible factor-1 (HIF-1) promotes its degradation by induction of HIF-alpha-prolyl-4-hydroxylases. The Biochemical journal.

[62] Shin DH, Chun YS, Lee DS, Huang LE, Park JW (2008). Bortezomib inhibits tumor adaptation to hypoxia by stimulating the FIH-mediated repression of hypoxia-inducible factor-1. Blood.

[63] Zhou Y, Liang QL, Ou WT, Liu QL, Zhang XN, Li ZY, Huang X (2014). Effect of stable transfection with PHD3 on growth and proliferation of HepG2 cells in vitro and in vivo. International journal of clinical and experimental medicine.

[64] Liang QL, Li ZY, Zhou Y, Liu QL, Ou WT, Huang ZG (2012). Construction of a recombinant eukaryotic expression vector containing PHD3 gene and its expression in HepG2 cells. Journal of experimental & clinical cancer research.

[65] Portolani N, Coniglio A, Ghidoni S, Giovanelli M, Benetti A, Tiberio GA, Giulini SM (2006). Early and late recurrence after liver resection for hepatocellular carcinoma: prognostic and therapeutic implications. Annals of surgery.

[66] Deng S, Zhang P, Zeng H, Wang W, Jin T, Wang J, Dong Q (2014). Factor-inhibiting hypoxia-inducible factor expression in patients with high-risk locally advanced renal cell carcinoma and its relationship with tumor progression. The Kaohsiung journal of medical sciences.

[67] Bruix J, Sherman M, Llovet JM, Beaugrand M, Lencioni R, Burroughs AK, Christensen E, Pagliaro L, Colombo M, Rodes J (2001). HCC EPoEo. Clinical management of hepatocellular carcinoma. Conclusions of the Barcelona-2000 EASL conference. European Association for the Study of the Liver. Journal of hepatology.

[68] Li WF, Ou Q, Dai H, Liu CA (2015). Lentiviral-Mediated Short Hairpin RNA Knockdown of MTDH Inhibits Cell Growth and Induces Apoptosis by Regulating the PTEN/AKT Pathway in Hepatocellular Carcinoma. International journal of molecular sciences.

[69] Chen J, Liu WB, Jia WD, Xu GL, Ma JL, Huang M, Deng YR, Li JS (2014). Overexpression of Mortalin in hepatocellular carcinoma and its relationship with angiogenesis and epithelial to mesenchymal transition. Int J Oncol.

[70] Liu X, Tan XL, Xia M, Wu C, Song J, Wu JJ, Laurence A, Xie QG, Zhang MZ, Liang HF, Zhang BX, Chen XP (2016). Loss of 11betaHSD1 enhances glycolysis, facilitates intrahepatic metastasis, and indicates poor prognosis in hepatocellular carcinoma. Oncotarget.

[71] Jia XQ, Zhang S, Zhu HJ, Wang W, Zhu JH, Wang XD, Qiang JF (2016). Increased Expression of PHGDH and Prognostic Significance in Colorectal Cancer. Translational oncology.

[72] Li JC, Yang XR, Sun HX, Xu Y, Zhou J, Qiu SJ, Ke AW, Cui YH, Wang ZJ, Wang WM, Liu KD, Fan J (2010). Up-regulation of Kruppel-like factor 8 promotes tumor invasion and indicates poor prognosis for hepatocellular carcinoma. Gastroenterology.

[73] Boddy JL, Fox SB, Han C, Campo L, Turley H, Kanga S, Malone PR, Harris AL (2005). The androgen receptor is significantly associated with vascular endothelial growth factor and hypoxia sensing via hypoxia-inducible factors HIF-1a, HIF-2a, and the prolyl hydroxylases in human prostate cancer. Clinical cancer research.

[74] Tsai MC, Chen KD, Wang CC, Huang KT, Wu CH, Kuo IY, Chen LY, Hu TH, Goto S, Nakano T, Dorling A, McVey JH, Chen CL (2015). Factor VII promotes hepatocellular carcinoma progression through ERK-TSC signaling. Cell death discovery.

[75] Feng X, Li H, Kornaga EN, Dean M, Lees-Miller SP, Riabowol K, Magliocco AM, Morris D, Watson PH, Enwere EK, Bebb G, Paterson A (2016). Low Ki67/high ATM protein expression in malignant tumors predicts favorable prognosis in a retrospective study of early stage hormone receptor positive breast cancer. Oncotarget.

