# Supplementary material for: Prolyl hydroxylase domain protein 3 and asparaginyl hydroxylase factor inhibiting HIF-1 levels are predictive of tumoral behavior and prognosis in hepatocellular carcinoma
Source: Oncotarget. 2017 Jan 16;8(8):12983–3002. doi: 10.18632/oncotarget.14677 (PMC5355071; doi:10.18632/oncotarget.14677)
Supplement: Supplementary file 1 [file oncotarget-08-12983-s001.pdf]

Prolyl hydroxylase domain protein 3 and asparaginyl hydroxylase factor inhibiting HIF-1 levels are predictive of tumoral behavior and prognosis in hepatocellular carcinoma

Supplementary Materials

Supplementary Table 1: Basic clinical features of 81 HCC patients

Parameters	Group	Patients number	Percentage (%)
Gender	Male	73	90.12
	Female	8	9.88
Age	≤ 50	45	55.56
	> 50	36	44.44
AFP (µg/l)	≤ 20	16	19.75
	> 20	65	80.25
GGT (U/l)	≤ 54	31	38.27
	> 54	50	61.73
ALT (ng/ml)	≤ 75	65	80.25
	> 75	16	19.75
HBV	Negative	9	11.11
	Positive	72	88.89
HCV	Negative	80	98.77
	Positive	1	1.23
Cirrhosis	No	10	12.35
	Yes	71	87.65
Tumor size (cm)	≤ 5	31	38.27
	> 5	50	61.73
Tumor encapsulation	None	34	41.98
	Complete	47	58.02
Tumor number	Single	61	75.31
	Multiple	20	24.69
Vascular invasion	No	52	64.20
	Yes	29	35.80
Distant metastasis	No	80	98.77
	Yes	1	1.23
Differentiation	I–II	56	69.14
	III–IV	25	30.86
TNM stage	I–II	43	53.09
	III–IV	38	46.91
BCLC stage	0 + A	45	55.56
	B + C	36	44.44
Adjuvant TACE	No	75	92.59
	Yes	6	7.41

Supplementary Table 2: Median score and percentage of higher expression cases of each protein in TAM

Target protein	Median score		Higher expression percentage	
	ANLTs	Tumors	ANLTs	Tumors
PHD1	4	4	46.90%	49.40%
PHD2	6	6	65.40%	60.50%
PHD3	8	6	85.20%	63.00%
FIH	12	6	95.10%	59.30%

Supplementary Table 3: Correlation between PHD3/FIH expression in TAMs and the clinicopathological characteristics in HCC patients (n = 81)

Parameters	Group	I	II	III	IV	Multiple Comparison		I vs IV#	
						χ^2	*p*-value	χ^2	*p*-value
Tumor size (cm)	≤ 5	18	3	7	3	6.995	0.0721		0.0209*
	> 5	18	9	8	15				
Tumor encapsulation	None	11	4	5	14	12.230	0.0066		**0.0014***
	Complete	25	8	10	4				
Vascular invasion	No	28	7	9	8	6.239	0.1006	6.000	0.0143
	Yes	8	5	6	10				
TNM stage	I–II	25	7	6	5	9.662	0.0217	8.438	**0.0037**
	III–IV	11	5	9	13				
BCLC stage	0 + A	25	7	7	6	6.930	0.0742	6.400	0.0114
	B + C	11	5	8	12				

[a]Group Ⅰ, Ⅱ, Ⅲ and Ⅳ refer to patients with $PHD3^H/FIH^H$, $PHD3^L/FIH^H$, $PHD3^H/FIH^L$ and $PHD3^L/FIH^L$ expression, respectively.

[*]Fisher exact test.

[#]Pairwise χ^2 test was applied with the Bonferroni correction. $P < 0.0083$ indicated significantly difference (0.0083 = 0.05/6).

Supplementary Table 4: Correlations among the expression levels of PHDs, FIH, MVD, Ki-67 and HIF-1 α*

Variable		HIF-1 α	Ki-67	MVD	FIH	PHD3	PHD2	PHD1
**PHD1**	r	0.103	-0.060	–0.014	0.216	**0.297**	**0.394**	-
	p	0.358	0.592	0.900	0.053	**0.007**	**< 0.001**	-
**PHD2**	r	**0.245**	0.070	–0.060	0.152	**0.269**	-	-
	p	**0.028**	0.534	0.596	0.175	**0.015**	-	-
**PHD3**	r	0.049	**–0.306**	–0.157	**0.301**	-	-	-
	p	0.664	**0.005**	0.162	**0.006**	-	-	-
**FIH**	r	0.172	**–0.259**	**–0.291**	-	-	-	-
	p	0.126	**0.020**	**0.008**	-	-	-	-
**MVD**	r	–0.084	0.042	-	-	-	-	-
	p	0.458	0.709	-	-	-	-	-
**Ki-67**	r	0.074	-	-	-	-	-	-
	p	0.510	-	-	-	-	-	-
**HIF-1 α**	r	-	-	-	-	-	-	-
	p	-	-	-	-	-	-	-

[*]Spearman's rank correlation test; r, Spearman's rank correlation coefficient.

Supplementary Table 5: Correlation between the PHD1 and PHD2 expression levels in TAMs and the clinicopathologic characteristics in HCC patients (*n* = 81)

Parameters	Total	PHD1		χ^2	*p*-value	PHD2		χ^2	*p*-value
		Low	High			Low	High		
**Gender**									
Male	73	36	37		0.712*	29	44		1.000*
Female	8	5	3			3	5		
**Age**									
≤ 50	45	21	24	0.632	0.427	14	31	2.986	0.084
> 50	36	20	16			18	18		
**AFP (μg/l)**									
≤ 20	16	7	9	0.376	0.540	10	6	4.411	**0.036**
> 20	65	34	31			22	43		
**GGT (U/l)**									
≤ 54	31	14	17	0.598	0.439	10	21	1.104	0.293
> 54	50	27	23			22	28		
**ALT (ng/ml)**									
≤ 75	65	34	31	0.376	0.540	26	39	0.034	0.855
> 75	16	7	9			6	10		
**HBV**									
Negative	9	3	6		0.312*	3	6		1.000*
Positive	72	38	34			29	43		
**HCV**									
Negative	80	41	39		0.494*	32	48		1.000*
Positive	1	0	1			0	1		
**Cirrhosis**									
No	10	5	5	0.002	0.967	5	5	0.526	0.468
Yes	71	36	35			27	44		
**Tumor size (cm)**									
≤ 5	31	14	17	0.598	0.439	12	19	0.013	0.908
> 5	50	27	23			20	30		
**Tumor encapsulation**									
None	34	22	12	4.653	**0.031**	14	20	0.068	0.794
Complete	47	19	28			18	29		
**Tumor number**									
Single	61	31	30	0.004	0.949	23	38	0.335	0.562
Multiple	20	10	10			9	11		
**Vascular invasion**									
No	52	24	28	1.158	0.282	20	32	0.066	0.797
Yes	29	17	12			12	17		
**Distant metastasis**									
No	80	41	39		0.494*	32	48		1.000*
Yes	1	0	1			0	1		
**Differentiation**									
I–II	56	28	28	0.028	0.868	26	30	3.638	0.056
III–IV	25	13	12			6	19		
**TNM stage**									
I–II	43	20	23	0.618	0.432	15	28	0.819	0.365
III–IV	38	21	17			17	21		
**BCLC stage**									
0 + A	45	21	24	0.632	0.427	18	27	0.010	0.919
B + C	36	20	16			14	22		
**Adjuvant TACE**									
No	75	36	39		0.201*	30	45		1.000*
Yes	6	5	1			2	4		
**MVD(per HPF)**									
< 45	46	23	23	0.016	0.899	17	29	0.290	0.590
≥ 45	35	18	17			15	20		
**Ki-67 LI**									
< 5%	32	15	17	0.296	0.586	14	18	0.399	0.528
≥ 5%	49	26	23			18	31		

*Fisher exact test; Ki-67 LI, Ki-67 labeling index.

Supplementary Table 6: Sequences of primers

Name of target gene	Sequence of primer
PHD1	Forward: 5′-CGTAAGGCACGTTGACAATC-3′<br>Reverse: 5′-ATGCACCTTAACGTCCCAGT-3′
PHD2	Forward: 5′-CAGCATGGACGACCTGATAC-3′<br>Reverse: 5′-TACATAACCCGTTCCATTGC-3′
PHD3	Forward: 5′-ATCGACAGGCTGGTCCTCTA-3′<br>Reverse: 5′-GATAGCAAGCCACCATTGC-3′
FIH	Forward: 5′-AAAATGTGGTTGGTTACGAAACAG-3′<br>Reverse: 5′-GACTCTATGTGATGCCACCAGTACA-3′
GAPDH	Forward: 5′-GGAGCGAGATCCCTCCAAAAT-3′<br>Reverse: 5′-GGCTGTTGTCATACTTCTCATGG-3′

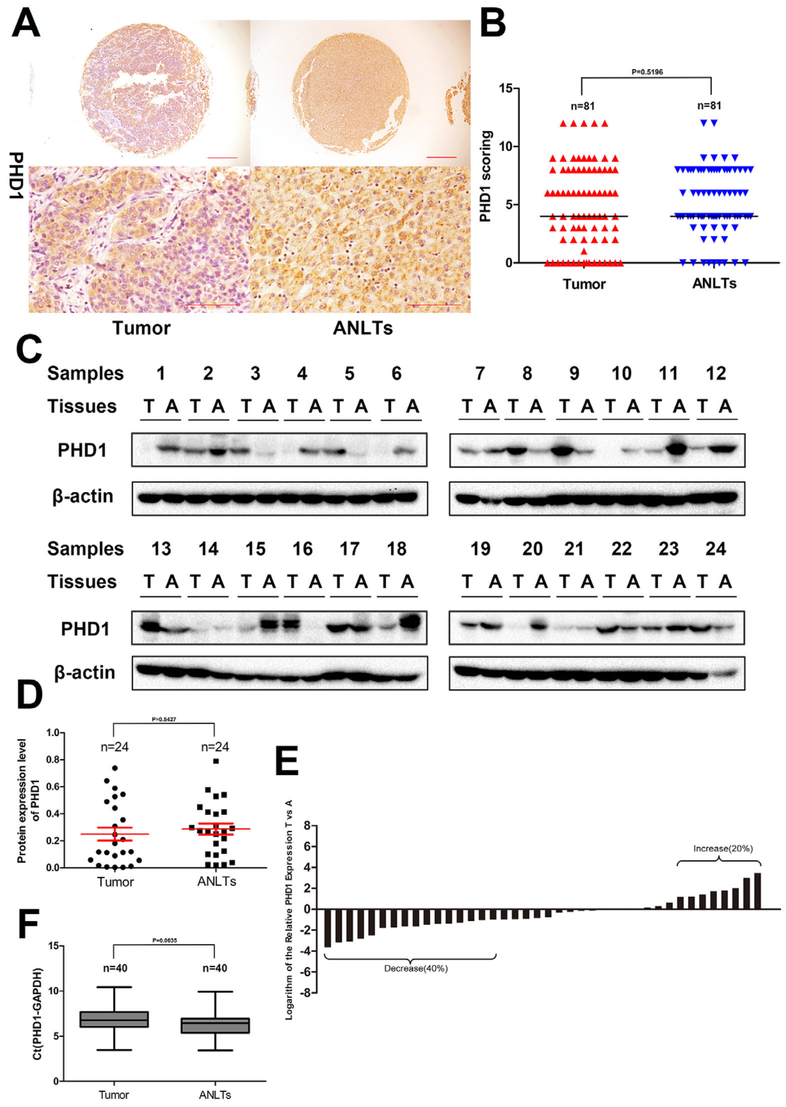

Supplementary Figure 1: Expression of PHD1 in HCC patients. (**A**) IHC analysis of PHD1 expression in 81 HCC tissues and paired ANLTs. Representative images were taken. Scale bar, 500 μm (upper) or 100 μm (lower). (**B**) IHC scoring was displayed by a scatter plot, with the median indicated. (**C**) Western blotting of tumor tissue and paired ANLTs in 24 randomly selected HCC cases with β actin as the loading control. (**D**) The relative densities of PHD1 protein in 24 cases were calculated and shown by a scatter plot, with the mean and SEM indicated. (**E**) the mRNA level of PHD1 was analysed by Real-time PCR in 40 randomly selected HCC tissues and paired ANLTs. The vertical axis means the logarithm base 2 of the relative expression of PHD1 in tumor tissue compared with ANLTs. Bar value ≤ -1 and ≥ 1 indicate that the expression of PHD1 is decreased and increased in tumors, respectively. (**F**) The ΔCt value was presented with a box plot reporting the median values and interquartile range.

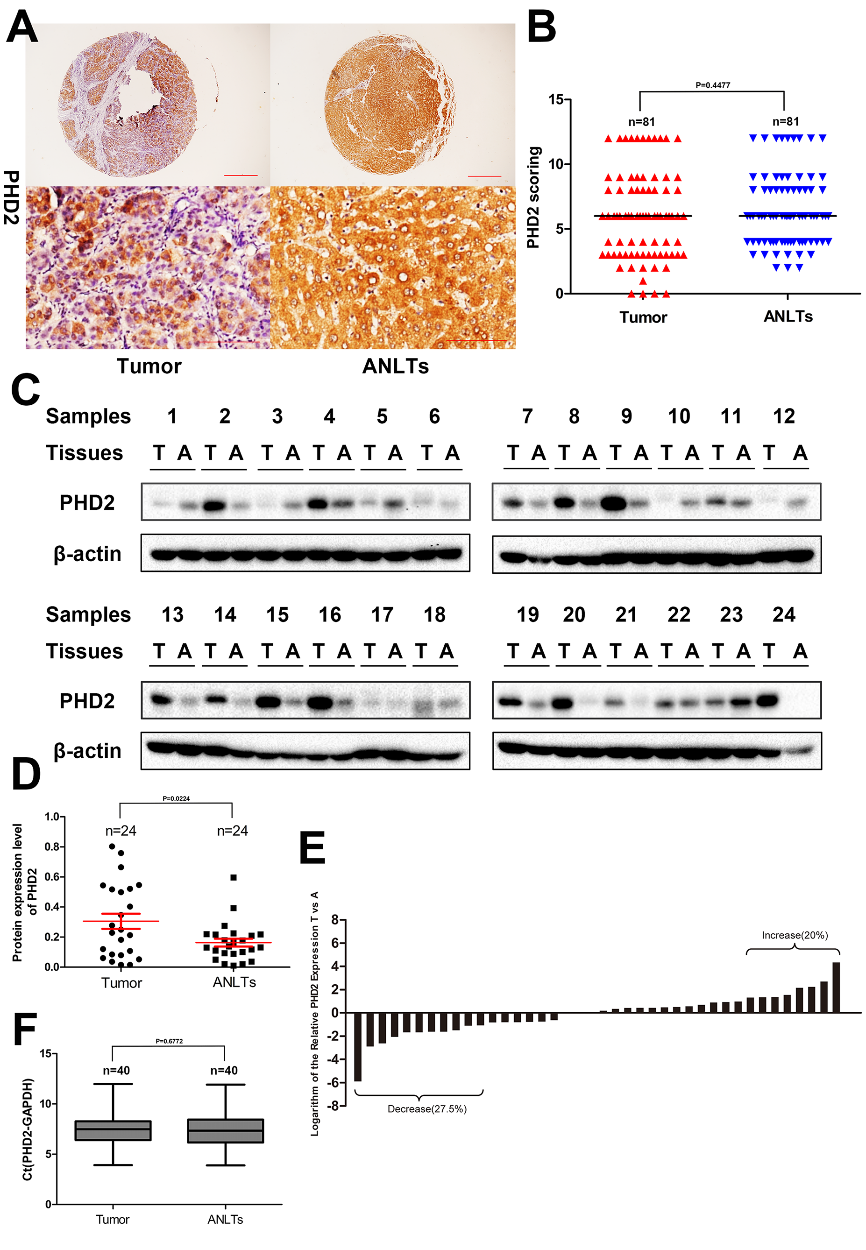

Supplementary Figure 2: Expression of PHD2 in HCC patients. (**A**) IHC analysis of PHD2 expression in 81 HCC tissues and paired ANLTs. Representative images were taken. Scale bar, 500 μm (upper) or 100 μm (lower). (**B**) IHC scoring was displayed by a scatter plot, with the median indicated. (**C**) Western blotting of tumor tissue and paired ANLTs in 24 randomly selected HCC cases with β actin as the loading control. (**D**) The relative densities of PHD2 protein in 24 cases were calculated and shown by a scatter plot, with the mean and SEM indicated. (**E**) the mRNA level of PHD2 was analysed by Real-time PCR in randomly selected 40 HCC tissues and paired ANLTs. The vertical axis means the logarithm base 2 of the relative expression of PHD2 in tumor tissue compared with ANLTs. Bar value ≤ -1 and ≥ 1 indicate that the expression of PHD2 is decreased and increased in tumors, respectively. (**F**) The ΔCt value was presented with a box plot reporting the median values and interquartile range.

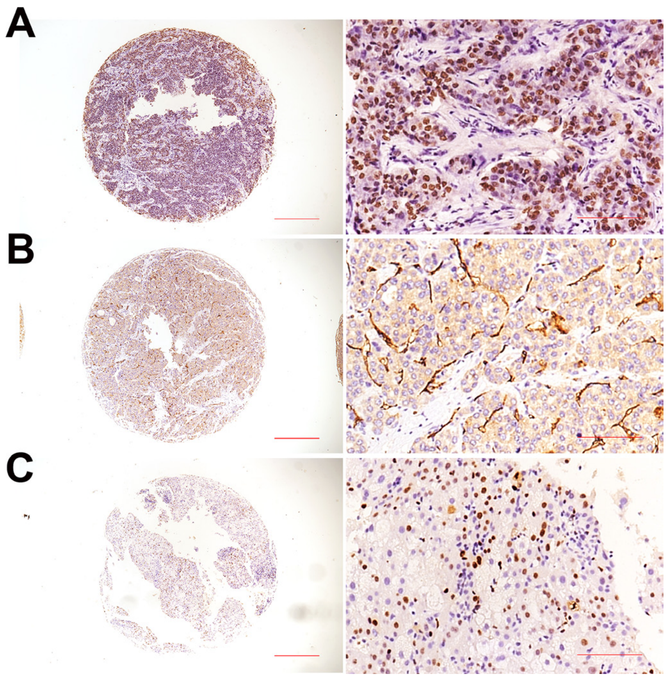

Supplementary Figure 3: Expression of HIF-1 α, CD34 and Ki-67 in HCC patients. (**A**) IHC analysis of HIF-1 α expression in 81 HCC tissues. Representative images were taken. Scale bar, 500 µm (left) or 100 µm (right). (**B**) IHC analysis of CD34 expression in 81 HCC tissues. Representative images were taken. Scale bar, 500 µm (left) or 100 µm (right). (**C**) IHC analysis of Ki-67 expression in 81 HCC tissues. Representative images were taken. Scale bar, 500 µm (left) or 100 µm (right).

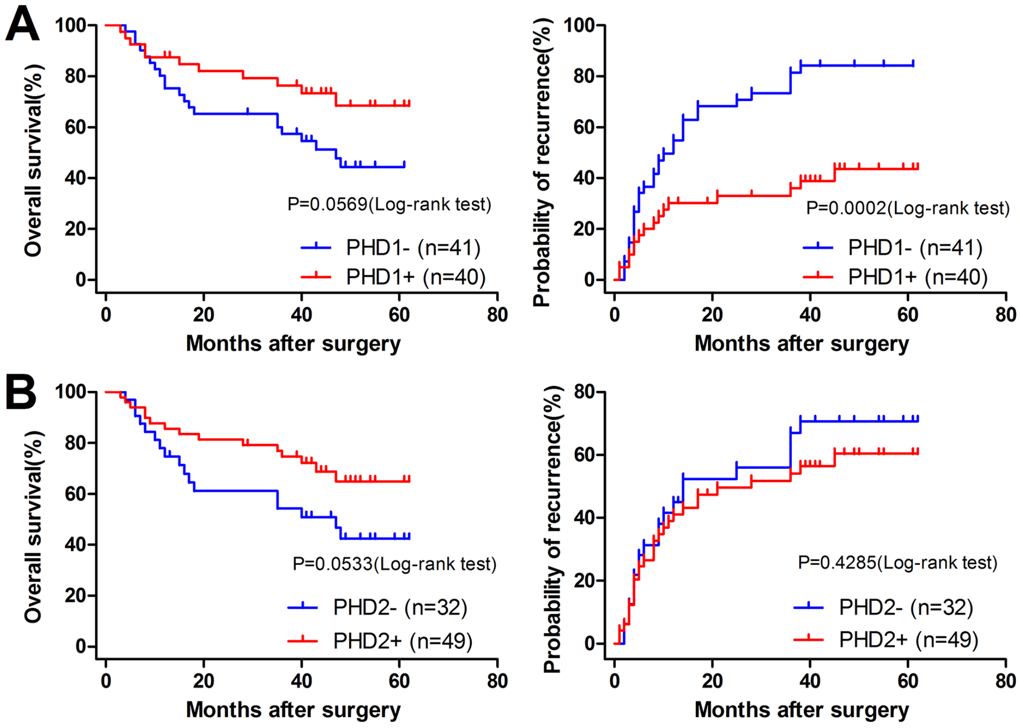

Supplementary Figure 4: Survival Kaplan-Meier curves for patients with different expression of PHD1 or PHD2 in TAMs. (**A**) OS and RR of the patients with different PHD1 expression in TAMs. OS 44.416% in PHD1(–) vs. 68.431% in PHD1(+), $p = 0.0569$. RR 84.089% in PHD1(–) vs. 43.565% in PHD1(+), $p = 0.0002$. (**B**) OS and RR of the patients with different PHD2 expression in TAMs. OS 42.409% in PHD2(–) vs. 64.879% in PHD2(+), $p = 0.0533$. RR 70.667% in PHD2(–) vs. 60.330% in PHD2(+), $p = 0.4285$. Log-rank test was used.

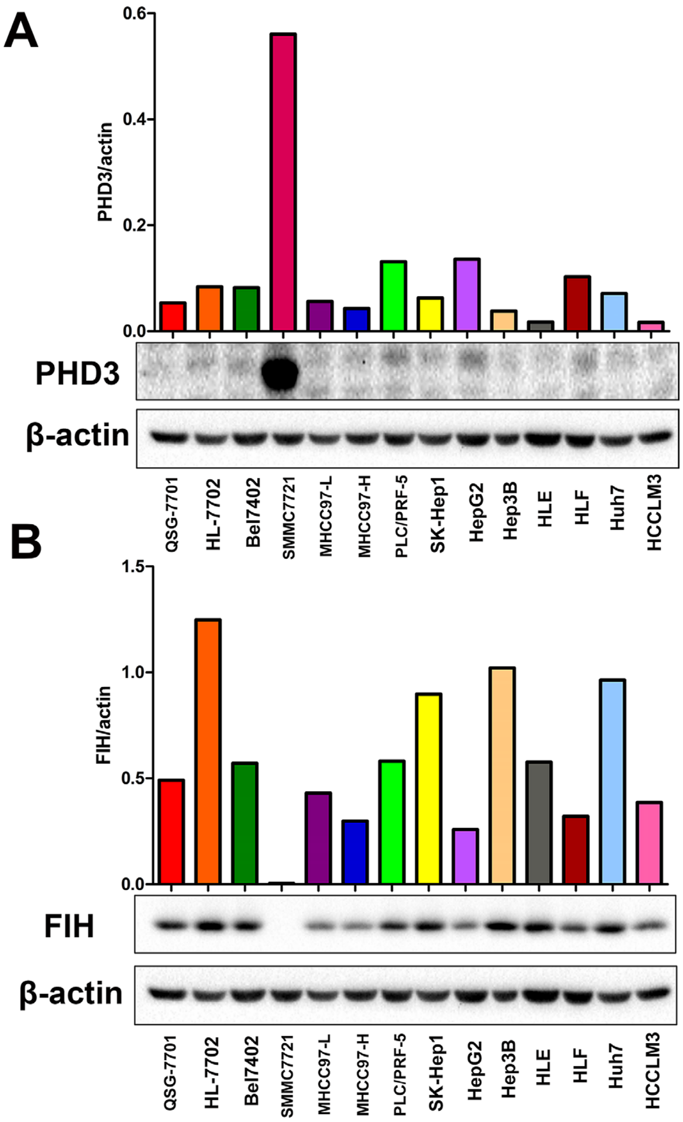

Supplementary Figure 5: Expression of PHD3 and FIH in different liver cell lines. (**A**) and (**B**) western blotting of PHD3 and FIH in two normal liver cell lines and 12 HCC cell lines, respectively. β-actin was the loading control. PHD3 was only obviously expressed in SMMC7721 cells, but was very weak or even undetectable in other cell lines. On the contrary, FIH could not be detected in SMMC7721 cells. The expression level of FIH varied in different cell lines with the highest level in normal liver cell HL-7702.
